# Allogeneic Serum and Macromolecular Crowding Maintain Native Equine Tenocyte Function in Culture

**DOI:** 10.3390/cells11091562

**Published:** 2022-05-05

**Authors:** Andrea Rampin, Ioannis Skoufos, Michael Raghunath, Athina Tzora, Nikolaos Diakakis, Nikitas Prassinos, Dimitrios I. Zeugolis

**Affiliations:** 1 Laboratory of Animal Science, Nutrition and Biotechnology, School of Agriculture, University of Ioannina, 47100 Arta, Greece; rampin88@gmail.com (A.R.); jskoufos@uoi.gr (I.S.); tzora@uoi.gr (A.T.); 2 School of Veterinary Medicine, Aristotle University of Thessaloniki, 54124 Thessaloniki, Greece; diakakis@vet.auth.gr (N.D.); ngreen@vet.auth.gr (N.P.); 3 Regenerative, Modular & Developmental Engineering Laboratory (REMODEL), Charles Institute of Dermatology, Conway Institute of Biomolecular & Biomedical Research, School of Mechanical & Materials Engineering, University College Dublin (UCD), D04 V1W8 Dublin, Ireland; 4 Center for Cell Biology and Tissue Engineering, Institute for Chemistry and Biotechnology, Zurich University of Applied Sciences, 8820 Wädenswil, Switzerland; ragh@zhaw.ch

**Keywords:** allogeneic serum, excluded volume effect, foetal bovine serum, serial passaging, tenocyte aging, tenocyte function

## Abstract

The absence of a native extracellular matrix and the use of xenogeneic sera are often associated with rapid tenocyte function losses during in vitro culture. Herein, we assessed the influence of different sera (equine serum and foetal bovine serum) on equine tenocyte morphology, viability, metabolic activity, proliferation and protein synthesis as a function of tissue-specific extracellular matrix deposition (induced via macromolecular crowding), aging (passages 3, 6, 9) and time in culture (days 3, 5, 7). In comparison to cells at passage 3, at day 3, in foetal bovine serum and without macromolecular crowding (traditional equine tenocyte culture), the highest number of significantly decreased readouts were observed for cells in foetal bovine serum, at passage 3, at day 5 and day 7 and without macromolecular crowding. Again, in comparison to traditional equine tenocyte culture, the highest number of significantly increased readouts were observed for cells in equine serum, at passage 3 and passage 6, at day 7 and with macromolecular crowding. Our data advocate the use of an allogeneic serum and tissue-specific extracellular matrix for effective expansion of equine tenocytes.

## 1. Introduction

Cell therapies require effective cell expansion in vitro to obtain sufficient numbers of functional cell populations. Unfortunately, current in vitro cell culture systems are associated with cell phenotypic drift and senescence as a function of serial passaging and therefore loss of the cells’ therapeutic potential [1,2,3,4,5]. Over the years, numerous studies have attributed this loss of native phenotype, function and therapeutic potential during ex vivo culture to the presence of xenogeneic serum [6] and/or the absence of a native extracellular matrix (ECM) [7,8,9,10].

In tendon engineering, tenocytes constitute the population of choice in cell-based therapies [11]. Over the years, numerous in vitro microenvironment modulators (e.g., topography [12,13], mechanical stimulation [14,15] and media supplements [16,17]) have been assessed as a means to maintain native tenocyte morphology (e.g., spindle-shaped) and physiological ECM synthesis (e.g., various collagen types and proteoglycans) in culture [18,19]. Unfortunately, bereft of their optimal tissue context, tenocytes rapidly lose their native function in vitro [20]. Surprisingly, the influence of serum and/or native ECM has been negated from traditional experimental approaches. Indeed, to the best of our knowledge, only one study has compared the effect of foetal bovine serum to serum-free conditions using tendon fibroblasts isolated from pregnant ewes and their foetuses [21]. Further, only one study has assessed the influence of different media (Dulbecco’s modified Eagle’s medium, Ham’s F12 nutrient mixture, RPMI 1640 medium, minimum essential medium with Earle’s salts, minimum essential medium with Hanks’ salts and Dulbecco’s modified Eagle’s medium/Ham’s F12 nutrient mixture) and different sera (foetal bovine serum, foetal equine serum and adult equine serum) in equine superficial digital flexor tendon explants [22]. With respect to the presence of native ECM, decellularised xenogeneic (the limited availability of autologous and/or allogeneic tendon tissues restricts their use) tendon tissues are used to either maintain tenocyte function [23,24] or to induce tenogenic differentiation [25,26], which, similarly to xenogeneic serum, harbour issues with interspecies disease transmission.

In human patients, approximately 30% of the musculoskeletal disorders (102.5 million reported musculoskeletal conditions in US alone [27]) are tendinopathy related [28], with the associated total mean estimated annual expenditure being EUR 840 per patient with Achilles tendinopathy [29]. In equine patients, musculoskeletal injuries account for 82% of all injuries (46% of them are tendon and ligament related) affecting racehorses competing in National Hunt and flat races [30] and over 70% of thoroughbred racehorse fatalities [31]. Considering the prevalence of tendon-related injuries and the associated healthcare expenditure in humans and the fatality rate in equine athletes, it is imperative to develop economic and scalable means to maintain tenocyte function in culture to allow for the development of therapeutic and reparative tenocyte-based interventions.

Herein we ventured to assess the influence of allogeneic (equine serum, ES) and xenogeneic (foetal bovine serum, FBS) sera and macromolecular crowding (MMC) in equine tenocyte (eTC) cultures, derived from superficial digital flexor tendons (SDFT), as a function of time (day 3, day 5, day 7) and aging (passage 3, passage 6, passage 9) in culture. eTC morphology, viability, metabolic activity, proliferation and protein synthesis were assessed and compared in both sera and at all time points and passages. Of particular importance is the use of MMC, which, based on the principles of excluded volume, restricts molecular diffusion and significantly accelerates the kinetics of biochemical reactions and biological processes [32,33,34,35,36,37]. In a similar manner, in a eukaryotic cell culture, MMC prohibits/restrains the diffusion of proteinases and procollagen, resulting in enhanced (up to 120-fold) and accelerated (within 2–6 days) collagen (and associated ECM) deposition [38,39,40]. It is worth noting that MMC has been shown to be far more effective than growth factor supplementation in human TC ECM deposition [41]. To validate this in eTC cultures, a range of tendon-specific ECM molecules were assessed (i.e., collagen type I, collagen type III, collagen type IV, collagen type V, collagen type VI, decorin, fibronectin and connexin).

## 2. Materials and Methods

### 2.1. Materials

Tissue culture plastics were purchased from Sarstedt (Nümbrecht, Germany). Equine SDFTs were collected from an abattoir and transferred on ice to the laboratory for cell extraction. Bovine collagen type I was used as the standard (Col. Std.) and was purchased from Symatese (Chaponost, France). Primary antibodies for immunofluorescence assays were purchased from Abcam (Cambridge, UK). Secondary antibodies were purchased from Thermo Fisher Scientific (Dublin, Ireland). All other reagents were purchased from Sigma Aldrich (Attica, Greece), unless otherwise stated.

### 2.2. Cell Isolation and Culture

eTCs were isolated from SDFTs as an explant culture. Briefly, paratenon and surrounding tissues were surgically removed. Tendon samples were washed in phosphate-buffered saline (PBS), cut in 5 mm × 5 mm × 3 mm pieces, placed at the centre of 6-well plates and allowed to adhere for 3 h at 37 °C in a humidified atmosphere of 5% CO_2_. Growth media containing high-glucose Dulbecco’s Modified Eagle Medium (DMEM), 1% penicillin/streptomycin (P/S) and 10% FBS or ES (TCS Biosciences, Buckingham, UK) were then added to the wells and the eTCs were allowed to migrate out of the tendon pieces. Once the cells reached confluence, they were detached from the culture plate using trypsin (0.05% trypsin in sterile filtered Hanks’ Balanced Salt Solution, HBSS, with 0.02% ethylenediaminetetraacetic acid tetrasodium salt and phenol red, incubated for 5 min at 37 °C in a humidified atmosphere of 5% CO_2_; porcine pancreas trypsin, 500–1200 BAEE U/mL (Sigma-Aldrich T3924, Schnelldorf, Germany) brought into a single cell suspension and then were either cryopreserved in the corresponding serum containing 10% dimethyl sulfoxide (DMSO) or plated at passage 1 for continuous expansion up to passage 9 in T75 tissue flasks. From passage 1 onwards, eTCs were passaged routinely having reached 75% confluence. At passages 3, 6 and 9, eTCs were plated at day 0 at a density of 10,000 cells/cm^2^ in 24-well (for SDS analysis), 48-well (for immunofluorescence, proliferation and viability analyses) or 96-well (for metabolic activity analysis) plates. After 24 h, growth media were switched to treatment media containing growth media supplemented with 100 μM L-ascorbic acid 2-phosphate sesquimagnesium salt hydrate (to induce collagen secretion) and 75 μg/mL carrageenan (CR, the MMC agent used to induce collagen and associated ECM deposition). Samples were analysed after 3, 5 and 7 days in culture.

### 2.3. Cell Morphology

Brightfield images were taken from cultures at each time point using an Olympus IX73P1F (Olympus Corporation, Tokyo, Japan) inverted fluorescence microscope, at a 20× magnification.

### 2.4. Cell Proliferation Analysis

At each time point, media were aspirated, and cells were washed for 10 min with HBSS at 37 °C in a humidified incubator of 5% CO_2_. Cell layers were fixed with 100 μL of 2% ice-cold paraformaldehyde (PFA) for 20 min, washed 3 times for 5 min with PBS, stained with 4′, 6-diamidino-2-phenylindole (DAPI) pre-diluted in methanol and further diluted in PBS for 5 min and washed 3 times for 5 min with PBS. Images were taken at 20× magnification with an Olympus IX73P1F (Olympus Corporation, Tokyo, Japan) inverted fluorescence microscope. Pictures were made binary with ImageJ (https://imagej.nih.gov/ij/ (accessed on 25 March 2022), NIH, Bethesda, MD, USA). Black nuclei were separated using the ImageJ (NIH, USA) watershed function and counted with the ‘particle count’ ImageJ (NIH, USA) plugin, using the following settings: particle size: 300-infinity (pixels); circularity: 0.3–1.0.

### 2.5. Cell Metabolic Activity Analysis

At each time point, cell layers were rinsed with HBSS and the cell layers were incubated with 440 μM resazurin in HBSS for 1 h at 37 °C in a humidified atmosphere of 5% CO_2_. Absorbance was read at 550 nm excitation and 595 nm emission with a SmartSpec Plus spectrophotometer (BioRad Laboratories, Hercules, CA, USA). The % of resazurin reduction was obtained using the following formula: RR% = (ALW − (AHW × CF)) × 100, where RR is the % of reduced resazurin, ALW is the absorbance at the lower wavelength, AHW is the absorbance at the higher wavelength and CF is the correlation factor, which is the ratio between the absorbance of resazurin at 550 nm and the absorbance of resazurin at 595 nm, which were measured as the difference between the absorbances of resazurin and HBSS alone at the two wavelengths.

### 2.6. Cell Viability Analysis

At each time point, eTCs were stained with 4 μM calcein-AM and 2 μM ethidium homodimer-1 in HBSS. After 30 min incubation at 37 °C in a humidified atmosphere of 5% CO_2_, the staining solution was replaced with HBSS. Three images per replicate were acquired using FITC and TRITC filters of an Olympus IX73P1F (Olympus Corporation, Tokyo, Japan) inverted fluorescence microscope at 10× magnification. Using ImageJ (NIH, USA) software, alive (calcein positive, green fluorescent) and dead (ethidium positive, red fluorescent) cells were counted from the corresponding fields. The average percentage of live cells to total cells was calculated.

### 2.7. Gel Electrophoresis Analysis

Sodium dodecyl sulphate polyacrylamide gel electrophoresis (SDS-PAGE) for collagen deposition analysis was conducted [42]. Briefly, at each time point, cell layers were washed with HBSS and subjected to a limited peptic digest for 2 h using 0.1 mg/mL pepsin from porcine gastric mucosa in 0.05 M acetic acid at room temperature. The reaction was stopped by adding 1 N NaOH. Samples were diluted 1 to 4 in double-distilled water (ddH_2_O), blended with 5× sample buffer and heat denatured at 95 °C for 5 min. Denatured samples were loaded onto 1-mm-thick gels (3% stacking and 5% separating acrylamide/bis 37.5:1 gels) and run under non-reducing conditions in an electrophoresis unit (Bio-Rad Laboratories, Watford, UK) at 50 V until the bromophenol band reached the end of the stacking gel (~30 min) and at 110 V until the bromophenol band reached the end of the separating gel (~60 min). The separating gel portions were briefly washed in ddH_2_O and fixed in 30% ethanol and 10% acetic acid overnight. Fixed gels were incubated for 1 h in the sensitising buffer (0.5% sodium thiosulfate, 6.8% sodium acetate, 0.125% glutaraldehyde and 30% ethanol), washed ×4 times for 12 min in ddH_2_O and incubated in 0.25% silver nitrate for 1 h (all at room temperature). Gels were then washed twice for 1 min in ddH_2_O and developed for 2 min in 2.5% sodium carbonate and 0.03% formaldehyde. The reaction was stopped with the addition of 1.46% ethylenediaminetetraacetic acid in ddH_2_O. The gels were scanned, and densitometry was performed on the acquired images using ImageJ (NIH, USA) software. The intensity of the α1(I) and α2(I) bands was normalised to the intensity of the α1(I) and α2(I) bands of the Col Std (1 mg/mL) and to the cell number (see Section 3.2).

### 2.8. Immunofluorescence Analysis

At each time point, cell layers were washed with PBS, fixed with 2% ice-cold PFA for 20 min, washed 3 times with PBS, blocked with 3% bovine serum albumin and incubated overnight at 4 °C with the following primary antibodies (all Abcam, Cambridge, UK): mouse monoclonal against collagen type I (ab90395, diluted 1:1000 in PBS), rabbit polyclonal against collagen type III (ab7778, diluted 1:200 in PBS), rabbit polyclonal against collagen type IV (ab6586, diluted 1:200 in PBS), rabbit polyclonal against collagen type V (ab7046, diluted 1:200 in PBS), rabbit polyclonal against collagen type VI (ab6588, diluted 1:200 in PBS), rabbit polyclonal against decorin (ab175404, diluted 1:200 in PBS), rabbit polyclonal against fibronectin (ab2413, diluted 1:200 in PBS) and rabbit polyclonal against connexin 43 (ab11370, diluted 1:200 in PBS). After 3 washes with PBS, a 30 min incubation was performed at room temperature with the following FITC-labelled secondary antibodies, diluted 1:500 in PBS: goat anti-rabbit IgG (A32731, Thermo Fisher Scientific, Dublin, Ireland) or goat anti-mouse IgG (A16091, Thermo Fisher Scientific, Dublin, UK). Cell layers were then washed 3 times with PBS and nuclei were stained with DAPI pre-diluted in methanol and further diluted in PBS. Samples were further washed 3 times before imaging with an Olympus IX73P1F (Olympus Corporation, Tokyo, Japan) inverted fluorescence microscope at 20× magnification. Collagen type I images were captured at 500 ms acquisition time, DAPI images were captured at 6.4 ms acquisition time and all the other images were captured at 72 ms acquisition time. Five images were captured per replicate. Negative controls (without primary antibody) were run in parallel. Fluorescence intensity was measured with ImageJ (NIH, USA) software and normalised on cell number.

### 2.9. Statistical Analysis

All results are presented as the mean ± standard deviation. Initial statistical analysis was performed using MINITAB™ software (Minitab Inc., State College, PA, USA). When the populations were of normal distribution (Anderson–Darling normality test) and had equal variance (Bartlett’s and Levene’s tests), a two-sample *t*-test was used for pairwise comparisons and ANOVA was used for multiple comparisons. When either of the parametric analysis assumptions were violated, non-parametric analysis was conducted, using Mann–Whitney tests for two-samples and Kruskal–Wallis tests for multiple comparisons. Secondary statistical analysis was conducted using Prism 8 for macOS (GraphPad Software LLC, San Diego, CA, USA). In this case, two-way ANOVA for multiple comparisons was carried out to compare all groups to cells at passage 3, at day 3 in FBS without MMC. Statistical significance, for all analyses, was accepted for *p* < 0.05.

## 3. Results

### 3.1. Cell Morphology and Growth Surface Coverage Analyses

Qualitative brightfield microscopy analysis (Supplementary Figure S1) revealed that cells, in general, at day 3, when they were still at low density, adopted a triangular and/or elongated morphology, whilst at day 7, when they were at high density, exhibited a spindle-shaped morphology. Further, in FBS at day 7, the cells were able to cover the entire growth surface, whilst in ES at day 7, they appeared to lose their capacity to cover the entire growth surface as a function of passaging. MMC did not affect the cell morphology or the capacity of cells to cover the growth surface.

### 3.2. Cell Proliferation, Metabolic Activity and Viability Analyses

In general, in FBS at all time points and independently of the absence or presence of MMC, cell proliferation was increased or remained constant as a function of passaging and time in culture, whilst in ES at all time points and independently of the absence or presence of MMC, cell proliferation was decreased as a function of passaging and was increased or remained constant as a function of time in culture for a given passage (Supplementary Figure S2 and Table S1).

Metabolic activity wise (Supplementary Figure S3 and Table S2), in both FBS and ES and across all passages, MMC only reduced metabolic activity at day 3. Metabolic activity at passage 9, in comparison to passage 3, was only increased for cells in FBS, −MMC at day 5 and day 7. In general, metabolic activity was increased or remained constant as a function of time in culture for a given passage.

With respect to cell viability in ES, all groups had cell viability > 95%, whilst in FBS, all groups had > 95% cell viability apart from cells at day 5, without MMC and passage 3 (79%), passage 6 (94%) and passage 9 (84%) and with MMC and passage 3 (88%); and at day 7 without MMC and passage 3 (87%), passage 6 (86%) and passage 9 (84%) (Supplementary Figure S4 and Table S3).

### 3.3. Gel Electrophoresis Analysis

**Figure 1:**
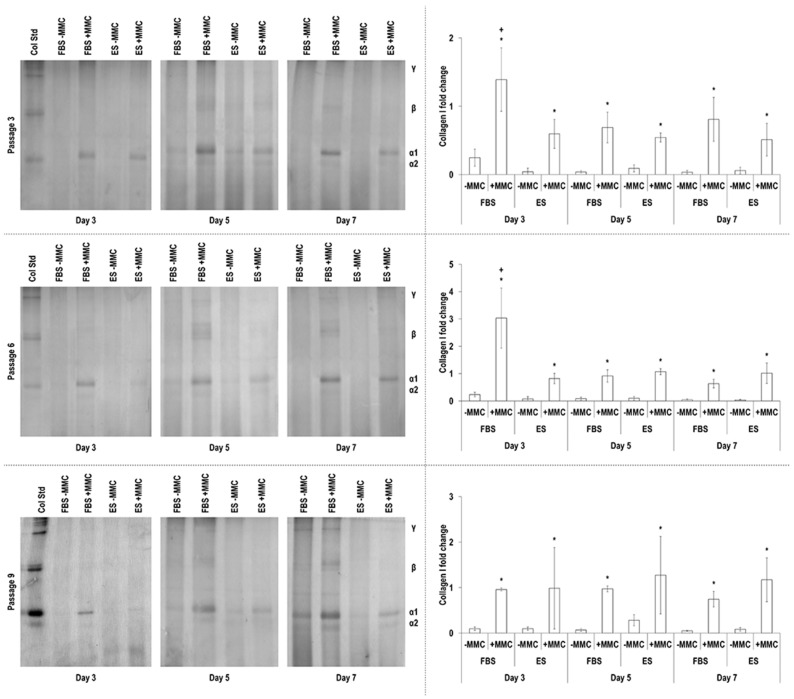
SDS-PAGE and densitometry analysis as a function of serum, passage, MMC and time in culture. + indicates the highest (*p* < 0.05) value at a given passage. * indicates significant difference between the −MMC and the +MMC at a given passage, serum and time point.

SDS-PAGE and densitometric analysis (Figure 1 and Supplementary Table S4) revealed that at all passages, all time points and independently of the serum used, MMC significantly (*p* < 0.05) increased collagen deposition and within the MMC groups, the highest (*p* < 0.05) collagen deposition was detected at day 3 in FBS at passage 3 and passage 6 cells.

### 3.4. Immunofluorescence Analysis

**Figure 2:**
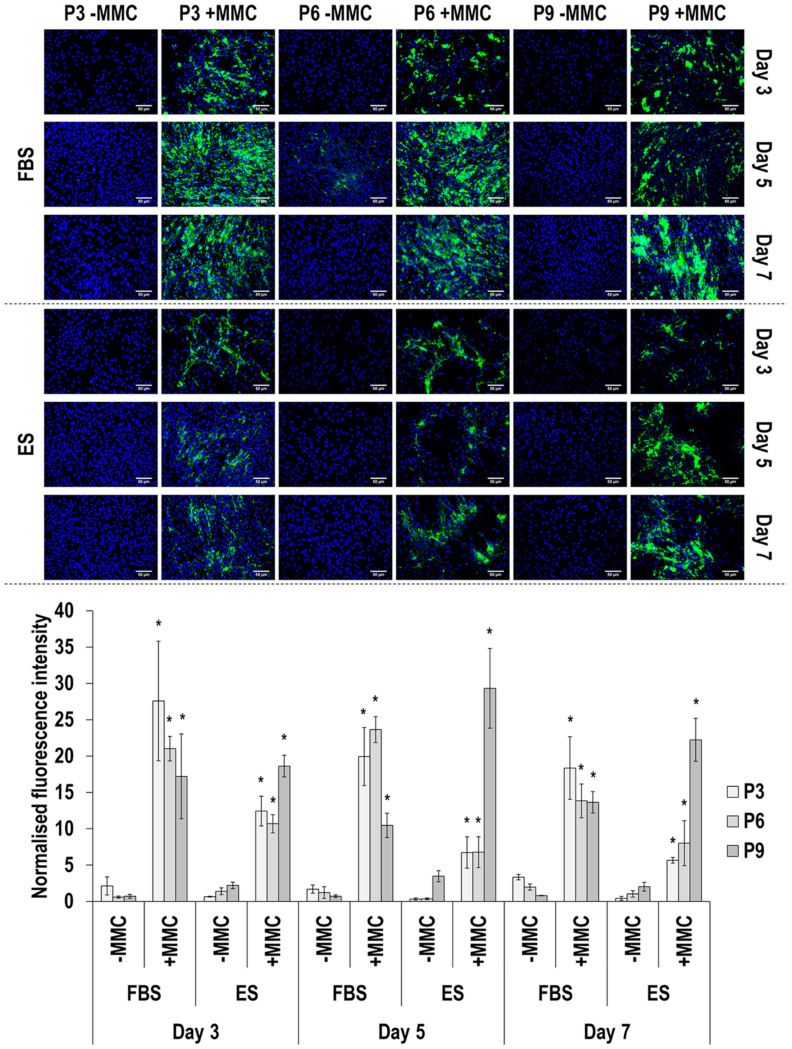
Immunofluorescence and image intensity analysis for collagen type I as a function of serum, passage, MMC and time in culture. Fluorescence intensity was normalised to cell number. * indicates a significant (*p* < 0.05) increase in comparison to cells at passage 3, day 3, in FBS and without MMC. Scale bar: 50 μm.

Collagen type I immunofluorescence and image intensity analysis (Figure 2 and Supplementary Table S5) revealed that MMC at all passages, in both sera and at all time points, significantly (*p* < 0.05) increased its deposition. In general, collagen type I deposition was decreased as a function of passaging in FBS and was increased as a function of passaging in ES. In general, collagen type I deposition also was decreased as a function of time in culture.

**Figure 3:**
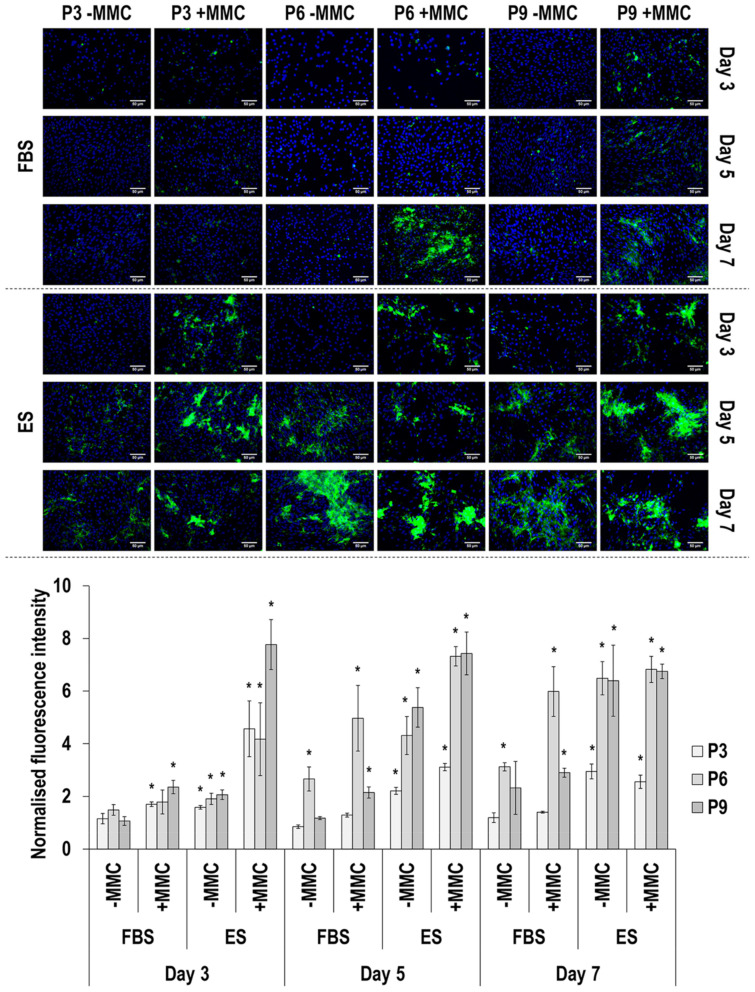
Immunofluorescence and image intensity analysis for collagen type III as a function of serum, passage, MMC and time in culture. Fluorescence intensity was normalised to cell number. * indicates a significant (*p* < 0.05) increase in comparison to cells at passage 3, day 3, in FBS and without MMC. Scale bar: 50 μm.

Immunofluorescence and image intensity analysis for collagen type III (Figure 3 and Supplementary Table S6) revealed that MMC significantly (*p* < 0.05) increased its deposition at day 3, in ES at all passages; at day 5, in FBS at passage 6 and in ES at all passages; and at day 7, only in FBS at passage 6. With respect to passaging, at day 3 only cells at passage 9 in ES with MMC deposited significantly (*p* < 0.05) higher amounts of collagen type III than cells at passage 3 and passage 6; at day 5, cells at passage 6 in FBS and without or with MMC and cells at passage 6 and passage 9 in ES and without or with MMC deposited significantly (*p* < 0.05) higher amounts of collagen type III than cells at passage 3 and passage 9 in FBS and passage 3 in ES; and at day 7, in both FBS and ES and without or with MMC, cells at passage 6 and passage 9 deposited significantly (*p* < 0.05) higher amounts of collagen type III than cells at passage 3. With respect to time in culture, the most obvious differences were that in FBS and in the presence of MMC, cells at passage 6 at day 7 deposited significantly (*p* < 0.05) more collagen type III than their counterparts at day 3; and in ES and in the absence of MMC, cells at all passages deposited significantly (*p* < 0.05) more collagen type III than their counterparts at day 3.

**Figure 4:**
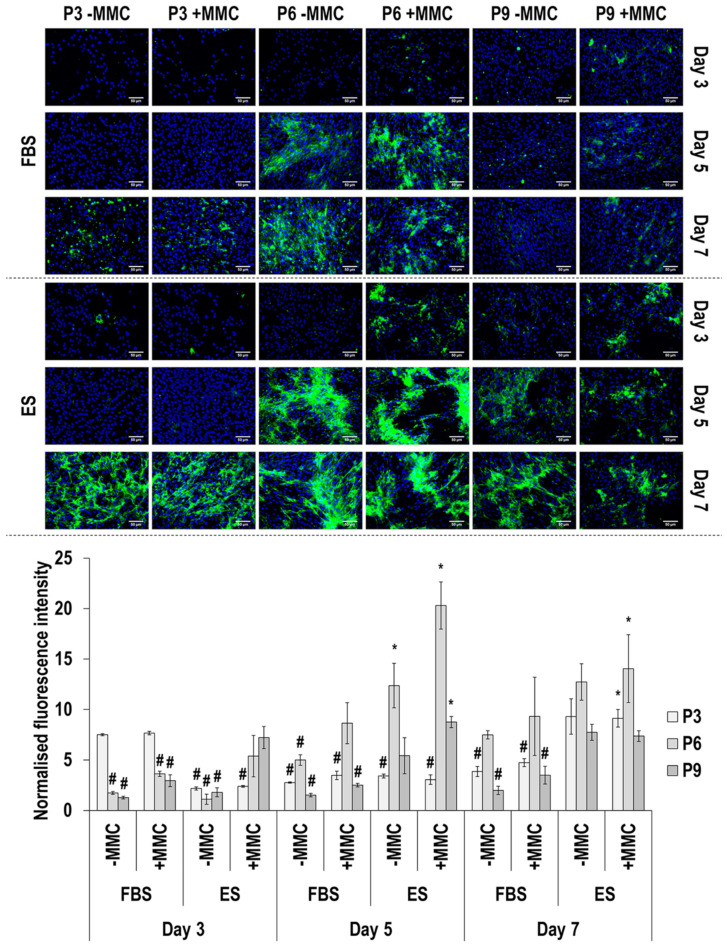
Immunofluorescence and image intensity analysis for collagen type IV as a function of serum, passage, MMC and time in culture. Fluorescence intensity was normalised to cell number. * indicates significant (*p* < 0.05) increase in comparison to cells at passage 3, day 3, in FBS and without MMC. # indicates a significant (*p* < 0.05) decrease in comparison to cells at passage 3, day 3, in FBS and without MMC. Scale bar: 50 μm.

Immunofluorescence and image intensity analysis for collagen type IV (Figure 4 and Supplementary Table S7) revealed that MMC significantly (*p* < 0.05) increased its deposition at day 3 and day 5 in FBS and ES at passage 6 and passage 9, but at day 7, it had no effect (*p* > 0.05). Passaging in FBS at day 3 resulted in significantly (*p* < 0.05) reduced collagen type IV deposition and at day 5 and day 7 resulted in significantly (*p* < 0.05) increased deposition from passage 3 to passage 6 and significantly (*p* < 0.05) decreased deposition from passage 6 to passage 9, independently on whether MMC was used. Passaging in ES at day 3 resulted in significantly (*p* < 0.05) increased collagen type IV deposition from passage 3 to passage 6 and passage 9, only when MMC was used, and at day 5 and day 7 resulted in significantly (*p* < 0.05) increased deposition from passage 3 to passage 6 and significantly (*p* < 0.05) decreased deposition from passage 6 to passage 9, independently on whether MMC was used. With respect to time in culture, in FBS at passage 3, collagen type IV deposition was significantly (*p* < 0.05) reduced from day 3 to day 7 and at passage 6 and passage 9, collagen type IV deposition was significantly (*p* < 0.05) increased from day 3 to day 7; all independently on whether MMC was used. In ES, time in culture, in general, resulted in significantly (*p* < 0.05) increased collagen type IV deposition, independently on whether MMC was used.

**Figure 5:**
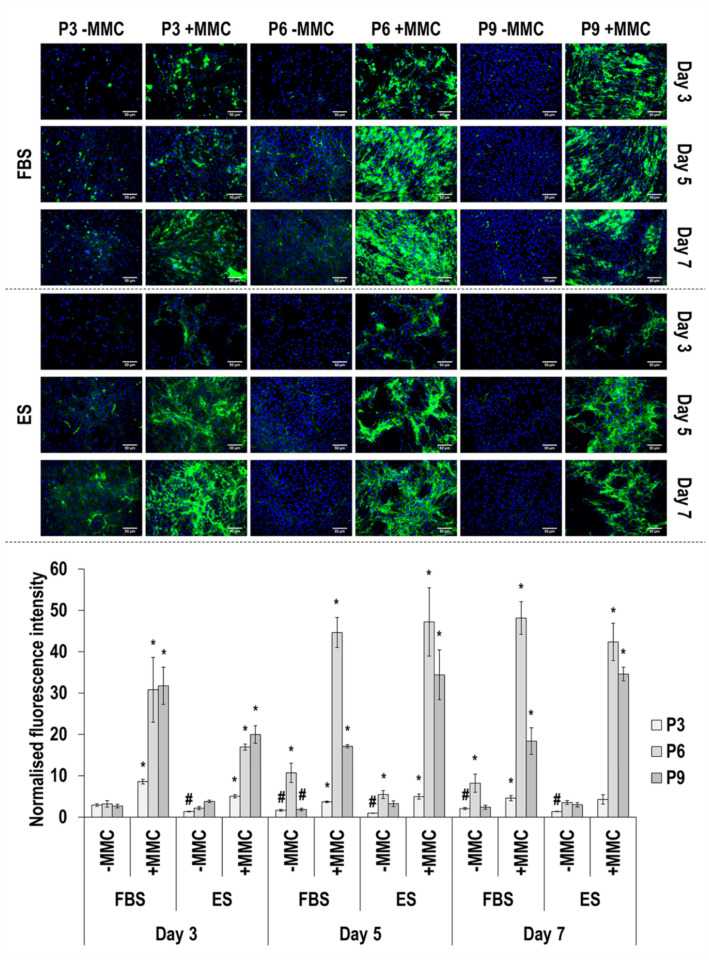
Immunofluorescence and image intensity analysis for collagen type V as a function of serum, passage, MMC and time in culture. Fluorescence intensity was normalised to cell number. * indicates a significant (*p* < 0.05) increase in comparison to cells at passage 3, day 3, in FBS and without MMC. # indicates significant (*p* < 0.05) decrease in comparison to cells at passage 3, day 3, in FBS and without MMC. Scale bar: 50 μm.

Immunofluorescence and image intensity analysis for collagen type V (Figure 5 and Supplementary Table S8) revealed that MMC significantly (*p* < 0.05) increased collagen type V deposition in all the examined conditions. In FBS without MMC, at day 3, passaging had no effect (*p* > 0.05) in collagen type V deposition and at day 5 and day 7, passage 6 induced the highest (*p* < 0.05) collagen type V deposition. In FBS with MMC, at day 3 passage 6 and passage 9 induced significantly (*p* < 0.05) higher collagen type V deposition than passage 3 and at day 5 and day 7, passage 6 induced significantly (*p* < 0.05) higher collagen type V deposition than passage 3 and passage 9 and passage 9 induced significantly (*p* < 0.05) higher collagen type V deposition than passage 3. In ES without and with MMC, at day 3, day 5 and day 7, passage 6 and passage 9 induced significantly (*p* < 0.05) higher collagen type V deposition than passage 3. With respect to time in culture, in FBS without MMC, collagen type V deposition was only significantly (*p* < 0.05) increased for passage 6, from day 3 to day 5. In FBS with MMC, collagen type V deposition was significantly (*p* < 0.05) decreased for passage 3 and passage 9 from day 3 to day 5 and day 7 and was significantly (*p* < 0.05) increased for passage 6 from day 3 to day 5 and day 7. In ES without MMC, collagen type V deposition was only significantly (*p* < 0.05) increased for passage 6, from day 3 to day 5. In ES with MMC, collagen type V deposition was significantly (*p* < 0.05) increased for passage 6 and passage 9, from day 3 to day 5 and day 7.

**Figure 6:**
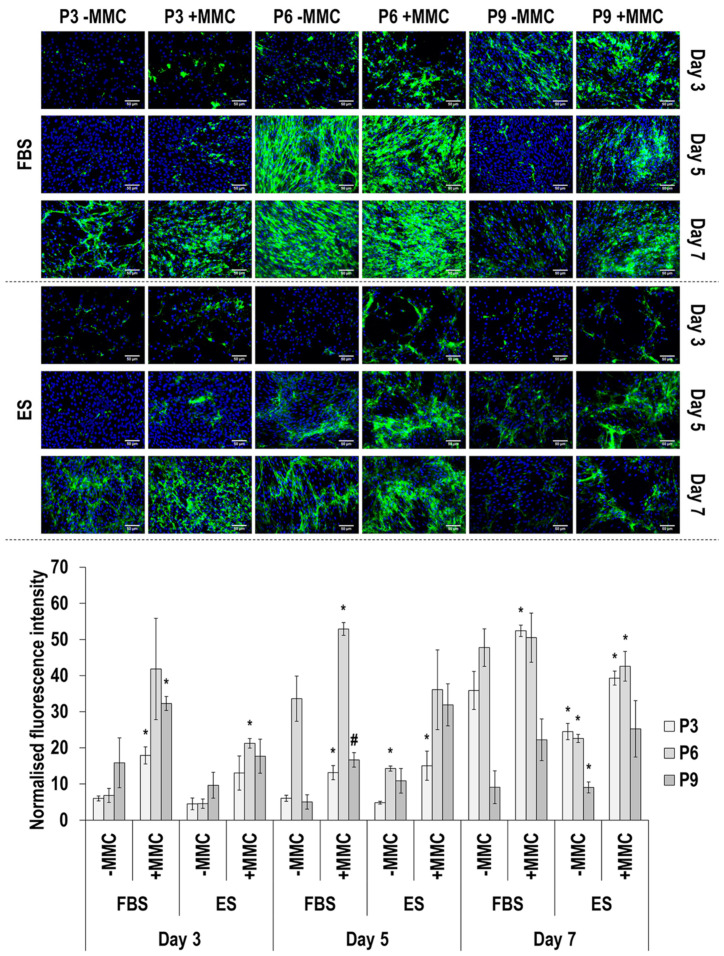
Immunofluorescence and image intensity analysis for collagen type VI as a function of serum, passage, MMC and time in culture. Fluorescence intensity was normalised to cell number. * indicates a significant (*p* < 0.05) increase in comparison to cells at passage 3, day 3, in FBS and without MMC. # indicates a significant (*p* < 0.05) decrease in comparison to cells at passage 3, day 3, in FBS and without MMC. Scale bar: 50 μm.

Immunofluorescence and image intensity analysis for collagen type VI (Figure 6 and Supplementary Table S9) made apparent that MMC significantly (*p* < 0.05) increased collagen type VI in all groups, but in passage 6, in FBS at day 7. In FBS without MMC, at day 3, passage 9 induced a significantly (*p* < 0.05) higher collagen type VI deposition than passage 3 and passage 6; at day 5, passage 6 induced significantly (*p* < 0.05) higher collagen type VI deposition than passage 3 and passage 9; and at day 7, passage 3 and passage 6 induced significantly (*p* < 0.05) higher collagen type VI deposition than passage 9. In FBS with MMC, at day 3, passage 6 and passage 9 induced significantly (*p* < 0.05) higher collagen type VI deposition than passage 3; at day 5, passage 6 induced significantly (*p* < 0.05) higher collagen type VI deposition than passage 3 and passage 9; and at day 7, passage 3 and passage 6 induced significantly (*p* < 0.05) higher collagen type VI deposition than passage 9. In ES without MMC, at day 3, passage 9 induced significantly (*p* < 0.05) higher collagen type VI deposition than passage 3 and passage 6; at day 5, passage 6 and passage 9 induced significantly (*p* < 0.05) higher collagen type VI deposition than passage 3; and at day 7, passage 3 and passage 6 induced significantly (*p* < 0.05) higher collagen type VI deposition than passage 9. In ES with MMC, at day 3, no significant (*p* > 0.05) differences in collagen type VI deposition were observed as a function of passage; at day 5, passage 6 and passage 9 induced significantly (*p* < 0.05) higher collagen type VI deposition than passage 3; and at day 7, passage 3 and passage 6 induced significantly (*p* < 0.05) higher collagen type VI deposition than passage 9. With respect to time in culture, in FBS without MMC, at passage 3, collagen type VI deposition was significantly (*p* < 0.05) increased from day 3 and day 5 to day 7; at passage 6, collagen type VI deposition was significantly (*p* < 0.05) increased from day 3 to day 5 and day 7; and at passage 9, no significant (*p* > 0.05) difference in collagen type VI deposition was detected as function of time in culture. In FBS with MMC, at passage 3, collagen type VI deposition was significantly (*p* < 0.05) increased from day 5 to day 7; at passage 6, no significant (*p* > 0.05) difference in collagen type VI deposition was detected as function of time in culture; and at passage 9, the highest (*p* < 0.05) collagen type VI deposition was detected at day 3. In ES without MMC, at passage 3, collagen type VI deposition was significantly (*p* < 0.05) increased from day 3 and day 5 to day 7; at passage 6, collagen type VI deposition was significantly (*p* < 0.05) increased from day 3 to day 5 and day 7; and at passage 9, no significant (*p* > 0.05) difference in collagen type VI deposition was detected as function of time in culture. In ES with MMC, at passage 3, the highest (*p* < 0.05) collagen type VI deposition was detected at day 7; and at passage 6 and passage 9, the lowest (*p* < 0.05) collagen type VI deposition was detected at day 3.

**Figure 7:**
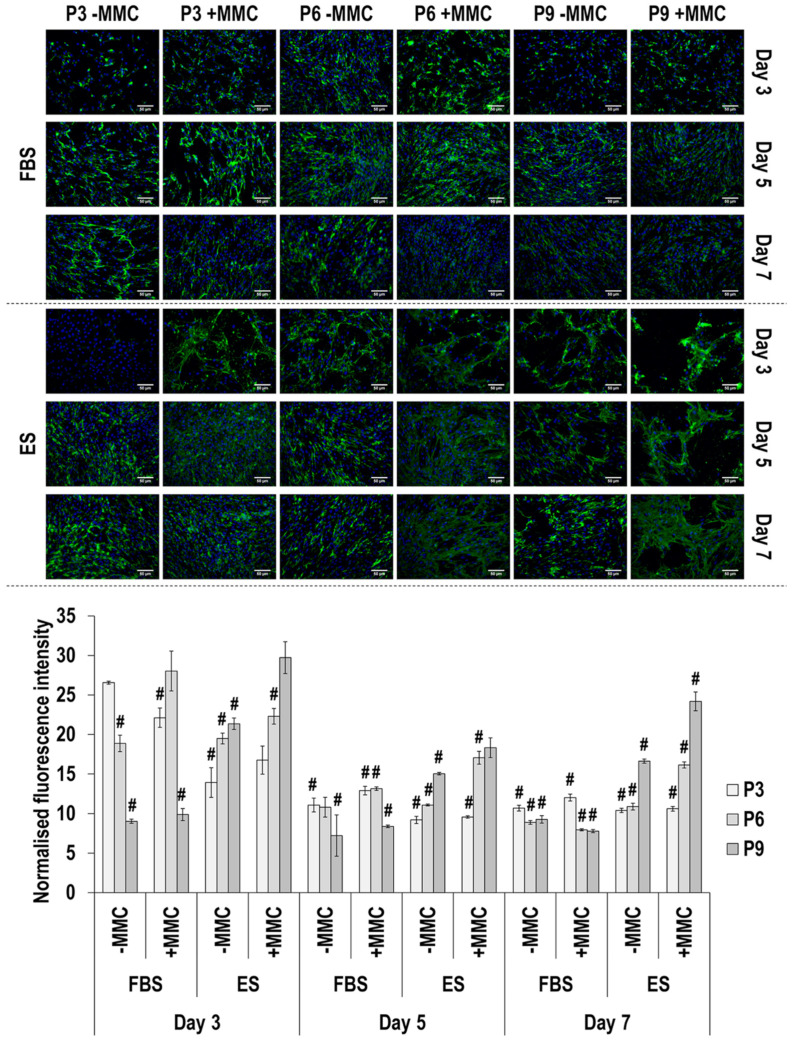
Immunofluorescence and image intensity analysis for fibronectin as a function of serum, passage, MMC and time in culture. Fluorescence intensity was normalised to cell number. # indicates a significant (*p* < 0.05) decrease in comparison to cells at passage 3, day 3, in FBS and without MMC. Scale bar: 50 μm.

Immunofluorescence and image intensity analysis for fibronectin (Figure 7 and Supplementary Table S10) made apparent that, in general, MMC did not affect (*p* > 0.05) fibronectin deposition. With respect to passaging, in FBS without MMC, only at day 3, passaging resulted in a significant (*p* < 0.05) decrease in fibronectin deposition. In FBS with MMC, at day 3 and day 5, passage 9 induced the lowest (*p* < 0.05) fibronectin deposition and at day 7, passage 3 induced the highest (*p* < 0.05) fibronectin deposition. In ES without MMC, at day 3, passage 3 induced the lowest (*p* < 0.05) fibronectin deposition and at day 5 and day 7, passage 9 induced the highest (*p* < 0.05) fibronectin deposition. In ES with MMC, at day 3, day 5 and day 7, passage 3 induced the lowest (*p* < 0.05) fibronectin deposition and at day 3 and day 7, passage 9 induced the highest (*p* < 0.05) fibronectin deposition. With respect to time in culture, in FBS without and with MMC, passage 3 and passage 6 induced the highest (*p* < 0.05) fibronectin deposition at day 3 and, in passage 9 with MMC, fibronectin deposition was significantly (*p* < 0.05) decreased at day 7, in comparison to day 3 and day 5. In ES without and with MMC, all passages induced the highest (*p* < 0.05) fibronectin deposition at day 3.

**Figure 8:**
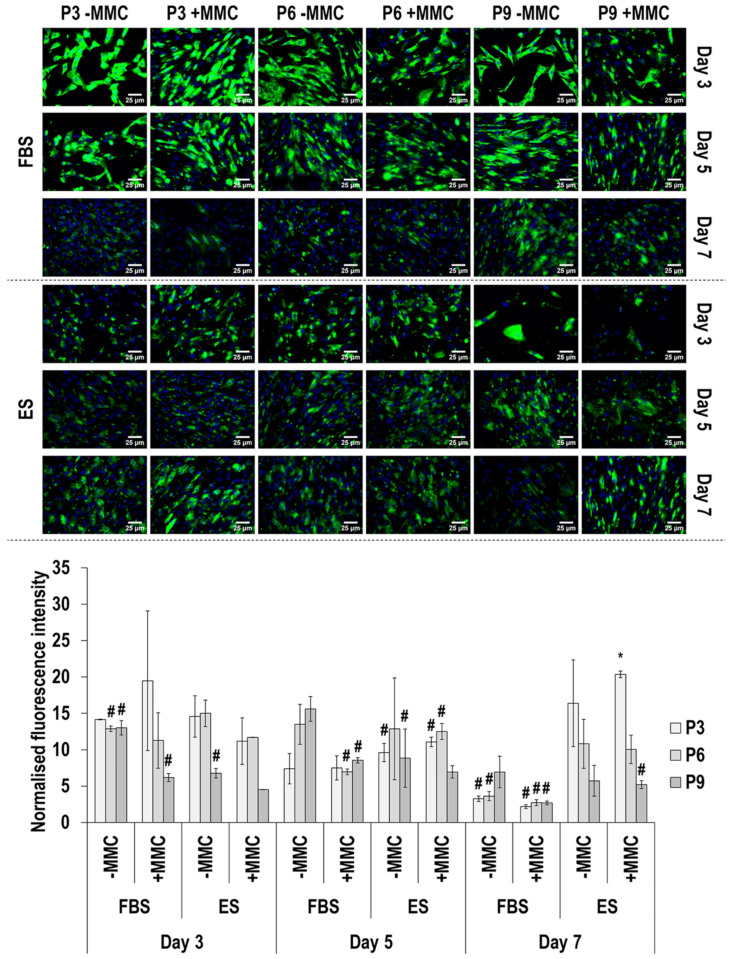
Immunofluorescence and image intensity analysis for decorin as a function of serum, passage, MMC and time in culture. Fluorescence intensity was normalised to cell number. * indicates a significant (*p* < 0.05) increase in comparison to cells at passage 3, day 3, in FBS and without MMC. # indicates a significant (*p* < 0.05) decrease in comparison to cells at passage 3, day 3, in FBS and without MMC. Scale bar: 25 μm.

Immunofluorescence and image intensity analysis for decorin (Figure 8 and Supplementary Table S11) revealed that MMC significantly (*p* < 0.05) reduced decorin expression at passage 9 in FBS at day 3; at passage 6 and passage 9 in FBS at day 5; and at passage 9 in FBS at day 7, whilst MMC had no effect (*p* > 0.05) in decorin expression in ES. With respect to passaging, in FBS without MMC, at day 3, no significant (*p* > 0.05) differences in decorin expression were identified as a function of passaging; at day 5, passage 3 induced the lowest (*p* < 0.05) decorin expression; and at day 7, passage 9 induced the highest (*p* < 0.05) decorin expression. In FBS with MMC, at day 3, passage 9 induced the lowest (*p* < 0.05) decorin expression; at day 5, and day 7, no significant (*p* > 0.05) differences in decorin expression were identified as a function of passaging. In ES without MMC, at day 3 and day 7, passage 9 induced the lowest (*p* < 0.05) decorin expression and at day 5, no significant (*p* > 0.05) differences in decorin expression were identified as a function of passaging. In ES with MMC, at day 3, day 5 and day 7, passage 9 induced the lowest (*p* < 0.05) decorin expression. With regards to time in culture, in FBS without and with MMC, day 7 induced the lowest (*p* < 0.05) decorin expression across all passages. In ES without MMC, decorin expression was significantly (*p* < 0.05) decreased at passage 6 from day 3 to day 5 and at passage 9 from day 3 to day 7 and with MMC, decorin expression was significantly (*p* < 0.05) increased at day 7 at passage 3 and at day 5 at passage 9; all compared with cells at day 3.

**Figure 9:**
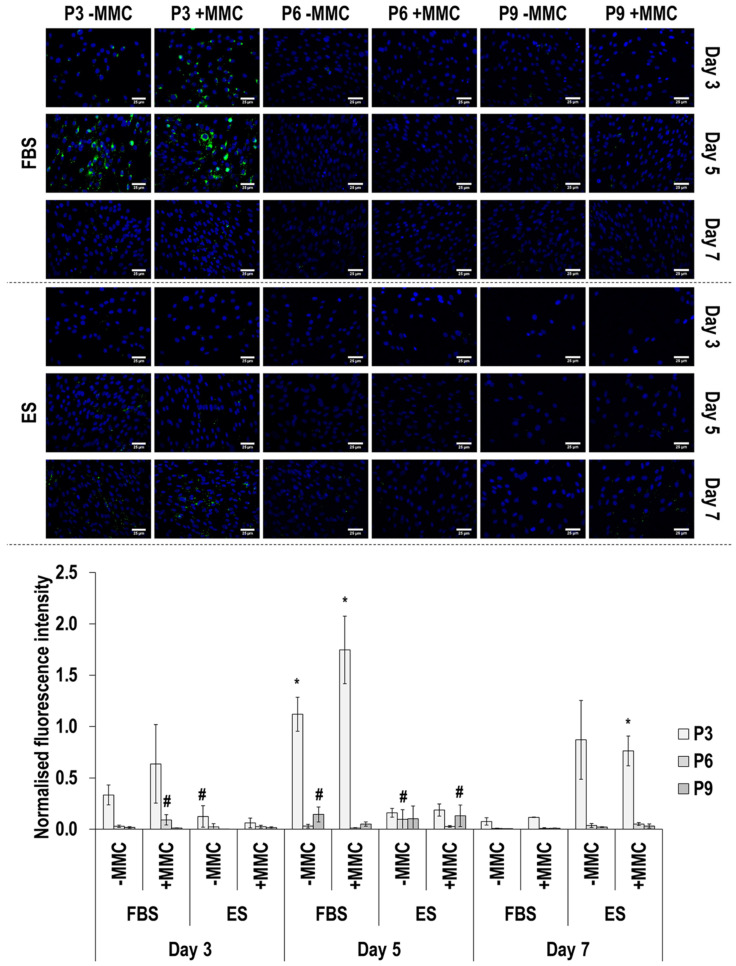
Immunofluorescence and image intensity analysis for connexin 43 as a function of serum, passage, MMC and time in culture. Fluorescence intensity was normalised to cell number. * indicates a significant (*p* < 0.05) increase in comparison to cells at passage 3, day 3, in FBS and without MMC. # indicates a significant (*p* < 0.05) decrease in comparison to cells at passage 3, day 3, in FBS and without MMC. Scale bar: 25 μm.

Immunofluorescence and image intensity analysis for connexin 43 expression (Figure 9 and Supplementary Table S12) revealed that, in general, MMC did not affect (*p* > 0.05) connexin 43 expression. With respect to passaging, apart from day 5 in ES without and with MMC, where no significant (*p* > 0.05) difference in connexin 43 expression was detected, in all other cases, connexin 43 expression was significantly (*p* < 0.05) higher in passage 3 than passage 6 and passage 9 in both FBS and ES. With respect to time in culture, in FBS without MMC, connexin 43 expression was significantly (*p* < 0.05) increased from day 3 to day 5 and significantly (*p* < 0.05) decreased from day 5 to day 7 at passage 3 and in FBS with MMC, connexin 43 was significantly (*p* < 0.05) decreased from day 5 to day 7 at passage 3. In ES without and with MMC, at passage 3, the highest (*p* < 0.05) connexin 43 expression was detected at day 7.

### 3.5. Comparison to Passage 3, in FBS, without MMC at Day 3

To assess the influence of passaging, time in culture, MMC and serum in eTCs, data were compared to eTCs at passage 3, at day 3, in FBS and without MMC (Supplementary Table S13). In comparison to eTCs at passage 3, day 3, in FBS and without MMC, the highest number of significantly decreased readouts were observed for eTCs at passage 3, day 5, in FBS and without MMC (metabolic activity, viability, collagen type I via SDS-PAGE and collagen type IV, collagen type V and fibronectin via immunofluorescence) and eTCs at passage 3, day 7, in FBS and without MMC (viability, collagen type I via SDS-PAGE and collagen type IV, collagen type V, fibronectin and decorin via immunofluorescence). In comparison to eTCs at passage 3, day 3, in FBS and without MMC (Supplementary Table S13), the highest number of significantly increased readouts were observed for eTCs at passage 3, day 7, in ES and with MMC (proliferation, metabolic activity, viability and collagen type I, collagen type III, collagen type IV, collagen type VI, decorin and connexin 43 via immunofluorescence) and eTCs at passage 6, day 7, in ES and with MMC (proliferation, metabolic activity, viability and collagen type I, collagen type III, collagen type IV, collagen type V and collagen type VI via immunofluorescence).

## 4. Discussion

TCs constitute the population of choice in tendon engineering [11]. Despite significant advances in ex vivo culture [18,19], TCs still readily lose their characteristic spindle-shaped morphology and protein synthesis capacity in vitro [20]. Although allogeneic serum [43,44,45,46] and tissue-specific ECM [47,48] have been advocated as potent cell function regulators, their combined effect in TC culture has yet to be evaluated. Herein, we assessed the influence of ES and FBS in eTC cultures, as a function of time in culture (day 3, day 5, day 7), passaging (passage 3, passage 6, passage 9) and MMC.

### 4.1. Basic Cell Function Analysis

Basic cell function analysis revealed that, in general, eTCs in ES at passage 9, although maintaining their viability, lost their spindle-shaped morphology and their capacity to cover the culture area and exhibited reduced proliferation and metabolic activity (all in comparison to eTCs in FBS). These observations are in accordance to previous publications, where equine bronchial fibroblasts in FBS, as opposed to ES, maintained better their morphology and needed shorter doubling and confluence times at increased passage number [49]. On the other hand, ES has shown beneficial effects over FBS as both a xenogeneic and allogeneic cell culture supplement. For example, dissociated mouse glial precursor cells, attached, proliferated and differentiated better in ES, as opposed to FBS-supplemented cultures [50], whilst ES exhibited marked superiority over FBS in equine monocyte-derived dendritic cell cultures [51]. With respect to proliferation, in general, at low passage, ES induced similar or higher proliferation than FBS, whilst at high passage, FBS induced higher proliferation than ES. In the literature, mixed observations have been reported. For example, FBS, as opposed to allogeneic serum, has been shown to decrease the proliferation of human synovium knee joint stem cells [52], whilst high concentrations (20% and 30% as opposed to 5% and 10%) of FBS have been shown to increase human adipose-derived stem cell proliferation during reprogramming [53]. Continuing on basic cellular function analysis, it is worth noting that freshly isolated foetal equine chondrocytes underwent similar modes of hypertrophy and physiological cell death in both FBS and ES, whilst chondrocytes from growing and mature horses did not undergo hypertrophy, neither in FBS nor in ES, and differences in pellet cultures from neonatal foals were observed in a serum-dependent manner [54]. These data may suggest a developmental stage-dependent cell response. We feel that there is not enough evidence in the literature to support this notion. For example, it has been suggested, due to poor regulation of FBS manufacturing, FBS may be contaminated with new-born and adult serum [55]. Further, in mouse astrocyte and human fibroblast cultures, adult bovine serum induced similar cell response to FBS, whilst in rat skeletal muscle cell line L6 cultures, the adult bovine serum induced higher proliferation and differentiation than FBS [56]. It is also worth noting that the beneficial effects of calf and adult bovine serum over FBS have also been documented in diploid (WI-38 and MRC-5) cell cultures [57]. We also ought to point out that attention should be paid to when sera are changed, as one study has shown deleterious effects on diploid (AG1522 and RbH2BO) cell cultures when FBS was replaced by new-born bovine, bovine calf or horse serum [58], whilst when FBS was replaced by ES in equine bone marrow stem cell cultures, the serum change did not affect cell viability, morphology and chondrogenic differentiation, but cells in FBS had shorter cell-doubling times, greater spontaneous bactericidal activity and secreted greater amounts of cytokines [59]. With respect to MMC supplementation, no differences were observed, which is in accordance to previous publications using human permanently differentiated [60] and stem [61] cell populations, as well as equine stem cells [62]. Collectively, these observations indicate a species-, tissue- and serum-dependent cell response.

### 4.2. SDS-PAGE and Immunofluorescence Analyses

Collagen type I is the most abundant ECM macromolecule of tendon tissue [63,64]. It is interestingly to note that in the absence of MMC, as evidenced by SDS-PAGE and immunofluorescence (in both sera, all passages and all time points), very little collagen type I deposition was observed. Although when human plantaris TCs where expanded in 10% FBS and then cultured in 1% FBS, strong bands of collagen type I were detected via Western blotting as early as 24 h in culture [65], in general, our data are in agreement with many publications with both human and equine TCs. For example, similarly to our observations, low collagen type I deposition has been reported previously in human hamstring TC cultures even after 14 days in culture using 10% FBS (highest collagen synthesis) or various serum-free, but growth factor-supplemented cultures [66] (one should note that the authors assessed collagen synthesis with Sirius red that largely over-estimates collagen content in serum containing media [67]). When human gracilis and/or semitendinosus TCs were cultured for 3 days in 10% and 1% FBS, again very little collagen was detected via Western blotting analysis, whilst collagen synthesis was increased as a function of increasing platelet-rich plasma concentration supplementation [68]. When human TCs in 10% FBS were cultured alone or in combination with IGF-1, PDGF-ββ, GDF-5 or TGFβ-3, almost no collagen was detected via SDS-PAGE and immunofluorescence after 13 days (substantial collagen deposition was detected with MMC and when MMC was coupled with TGFβ-3) [41]. When equine superficial digital flexor TCs from young (4.4 ± 1.7 years) and old (18 ± 2.4) donors were cultured for 28 days in high density (1.5 × 10^6^ cells/mL) within a fibrin system in 10% FBS and 0.02 μg/mL human recombinant TGFβ-3, the cells from the young donors (0.31 ± 0.18% collagen/mg dry weight) synthesised a lot lower collagen than the cells from the old donors (1.66 ± 0.8% collagen/mg dry weight) [69]. Collectively, all these data clearly illustrate a rather slow collagen synthesis and deposition in both human and equine TC cultures.

Collagen type I deposition was drastically enhanced by carrageenan supplementation, the MMC agent used herein, which aligns with previous observations in a diverse range of cell populations [70,71,72], including human TCs [41,73,74,75] and eTCs [76]. FBS maintained a constant intensity and a uniform distribution in collagen type I deposition up to passage 9; on the contrary, reflecting the reduction in cell proliferation and the formation of cell clusters, collagen type I total deposition was reduced and showed a mottled distribution at passage 9 in the presence of ES. Collagen type III is a crucial regulator of the diameter of collagen bundles during embryonic fibrillogenesis [77] and increased deposition is associated with scar tissue formation and inferior physical properties in the adult tendon [78]. It is also worth noting, that increased collagen type I to collagen type III ratio is indicative of scarring and aging [79,80,81,82]. Considering that almost in all cases the ratio of collagen type I to collagen type III was higher in FBS cultures (Supplementary Table S14), we believe that ES enabled physiological ECM synthesis, whilst FBS may have activated fibrotic pathways. To substantiate this, one should consider that serum-free conditions, as opposed to FBS conditions, have been shown to enhance the immunosuppressive and antifibrotic abilities of mesenchymal stem cells [83,84]. An alternative, and more probable, in our opinion, theory could be that the use of allogeneic (i.e., ES), as opposed to xenogeneic (i.e., FBS), serum activated the reparative, as opposed to proinflammatory, pathways. After all, previous studies have shown, for example, (a) allogeneic adipose-derived stem cells to induce cytotoxicity and faster cell death after exposure to xenogeneic serum [85]; and (b) FBS to induce increased production TNF-α (pro-inflammatory cytokine) and decreased production of IL-10 (anti-inflammatory cytokine) in equine monocyte cultures [86]. Collagen type IV deposition was not particularly affected by MMC supplementation; previous studies have shown MMC to increase (e.g., human TCs [75] and bone marrow stem cells [74]) or not affect (e.g., human TCs and dermal fibroblasts [74], eTCs [76]) collagen type IV deposition. In general, higher levels of collagen type IV were observed in the presence of ES, suggestive of basement membrane epithelium formation that is required for cell retention and the prevention of adhesion formation [87]. The marked increase observed in the presence of MMC in the deposition of collagen type V, a fibrillar collagen reported to play a role in tendinous fibril nucleation [88], is in line with previous observations in human TC [74,75] and eTC [76] cultures. Although no particular differences were observed between the two sera at passage 3, in ES, collagen type V total deposition followed the general reducing trend as a function of increasing passaging number as a direct consequence of reduced cell number. Collagen type VI is involved in cell migration and survival [89,90]. In accordance with previous publications with human TCs [74,75] and eTCs [76], MMC supplementation significantly increased collagen type VI deposition in most time points and culture conditions, although to a lesser extent than collagen type I; for example, its assembly requires a single extracellular proteolytic cleavage [91]. Again, FBS resulted in higher collagen type VI deposition at passages 6 and passage 9, possibly due to the reduced proliferation of eTCs in ES in these passages. Fibronectin, an essential ECM molecule for normal collagen deposition and organisation [92,93], deposition was steady throughout the culture period and was not affected by MMC. In previous studies with human TCs, fibronectin deposition was either reduced at early time points or was not affected at late time points [75] and with eTCs was not affected [76]. Although fibronectin deposition and organisation may be affected by serum containing fibronectin [94,95,96,97], its uneven distribution in ES at passage 9 is attributed to the reduced cell proliferation and the formation of cell clusters. Decorin is a small leucine-rich proteoglycan involved in skin and tendon collagen fibrillogenesis [98] and it is customarily used as a tenogenic marker [18,19]. We observed that decorin was invariably less expressed in day 7 compared to day 3 in the presence of FBS, whilst the opposite was the case for ES, especially in the presence of MMC, suggestive of effective native eTC function maintenance. Connexin 43, a gap-junction-associated protein with crucial function in tendon and tendon enthesis formation, function and response to loading [99,100], was observed at passage 3 and at day 3 and day 5 in FBS (higher synthesis was detected under MMC conditions), and in the presence of ES, at passage 3 and at day 7 (MMC independent synthesis). The decreased synthesis of connexin 43 as a function of time in culture (in FBS) and as function of passaging (in both FBS and ES) may be attributed to the absence of mechanical stimulation in this work, which coincides with previous publications that have shown a mechanical loading regime-, time in culture- and age- dependent connexin 43 synthesis and/or expression [101,102,103,104].

### 4.3. Comparison to Passage 3, in FBS, without MMC at Day 3

To assess the influence of passaging, time in culture, MMC and serum in eTC culture, we compared all groups to cells at passage 3, at day 3, in FBS and without MMC (essentially, the earliest passage the earliest time point and most conventional culture condition). One cannot but note that the highest number of significantly decreased readouts were observed for cells at passage 3, day 5 and day 7, in FBS and without MMC and the highest number of significantly increased readouts were observed for cells at passage 3 and passage 6, day 7, in ES and with MMC. These observations are in agreement with previous publications on the sensitivity of TCs in ex vivo culture in the presence of FBS [20]. They further advocate the use of allogeneic serum, when it is combined with tissue-specific ECM (in this case induced via MMC supplementation), to maintain TC phenotype. It is worth noting that MMC, in particular when combined with other in vitro microenvironment modulators (e.g., mechanical stimulation [74], oxygen tension [73], growth factor supplementation [41], surface topography and substrate rigidity [75]), has been shown to maintain TC phenotype in culture. Overall, our data mirror concerns associated with the use of xenogeneic serum in cell culture [105,106].

## 5. Conclusions

Bereft of their optimal tissue context and during in vitro culture, tenocytes readily lose their function. Our data (cell morphology, viability, metabolic activity, proliferation and protein synthesis analyses) indicate that the use of xenogeneic serum (i.e., foetal bovine serum in equine tenocyte cultures) and the lack of a tissue-specific extracellular matrix are the major culprits (lost their characteristics within 3 passages). In contrast, allogeneic serum (i.e., equine serum in equine tenocyte cultures) and a tissue-specific extracellular matrix (induced via macromolecular crowding) can prolong their lifespan in culture (up to 6 passages). These data further reinforce the notion for alternatives of foetal bovine serum in eukaryotic cell culture.

## Data Availability

The raw and processed data required to reproduce these findings are available on request from A.R.

## Supplementary Materials

The following supporting information can be downloaded at: https://www.mdpi.com/article/10.3390/cells11091562/s1, Table S1: eTC proliferation analysis as a function of macromolecular crowding (MMC), serum (foetal bovine serum, FBS; equine serum, ES), passage (P; 3, 6, 9) and days (D; 3 vs. 5, 3 vs. 7, 5 vs. 7) in culture. Table S2: eTC metabolic activity analysis as a function of macromolecular crowding (MMC), serum (foetal bovine serum, FBS; equine serum, ES), passage (P; 3, 6, 9) and days (D; 3 vs. 5, 3 vs. 7, 5 vs. 7) in culture. Table S3: eTC viability analysis as a function of macromolecular crowding (MMC), serum (foetal bovine serum, FBS; equine serum, ES), passage (P; 3, 6, 9) and days (D; 3 vs. 5, 3 vs. 7, 5 vs. 7) in culture. Table S4: eTC SDS-PAGE densitometry analysis as a function of macromolecular crowding (MMC), serum (foetal bovine serum, FBS; equine serum, ES), passage (P; 3, 6, 9) and days (D; 3 vs. 5, 3 vs. 7, 5 vs. 7) in culture. Table S5: eTC collagen type I immunofluorescence image intensity analysis as a function of macromolecular crowding (MMC), serum (foetal bovine serum, FBS; equine serum, ES), passage (P; 3, 6, 9) and days (D; 3 vs. 5, 3 vs. 7, 5 vs. 7) in culture. Table S6: eTC collagen type III immunofluorescence image intensity analysis as a function of macromolecular crowding (MMC), serum (foetal bovine serum, FBS; equine serum, ES), passage (P; 3, 6, 9) and days (D; 3 vs. 5, 3 vs. 7, 5 vs. 7) in culture. Table S7: eTC collagen type IV immunofluorescence image intensity analysis as a function of macromolecular crowding (MMC), serum (foetal bovine serum, FBS; equine serum, ES), passage (P; 3, 6, 9) and days (D; 3 vs. 5, 3 vs. 7, 5 vs. 7) in culture. Table S8: eTC collagen type V immunofluorescence image intensity analysis as a function of macromolecular crowding (MMC), serum (foetal bovine serum, FBS; equine serum, ES), passage (P; 3, 6, 9) and days (D; 3 vs. 5, 3 vs. 7, 5 vs. 7) in culture. Table S9: eTC collagen type VI immunofluorescence image intensity analysis as a function of macromolecular crowding (MMC), serum (foetal bovine serum, FBS; equine serum, ES), passage (P; 3, 6, 9) and days (D; 3 vs. 5, 3 vs. 7, 5 vs. 7) in culture. Table S10: TC fibronectin immunofluorescence image intensity analysis as a function of macromolecular crowding (MMC), serum (foetal bovine serum, FBS; equine serum, ES), passage (P; 3, 6, 9) and days (D; 3 vs. 5, 3 vs. 7, 5 vs. 7) in culture. Table S11: TC decorin immunofluorescence image intensity analysis as a function of macromolecular crowding (MMC), serum (foetal bovine serum, FBS; equine serum, ES), passage (P; 3, 6, 9) and days (D; 3 vs. 5, 3 vs. 7, 5 vs. 7) in culture. Table S12: eTC connexin 43 immunofluorescence image intensity analysis as a function of macromolecular crowding (MMC), serum (foetal bovine serum, FBS; equine serum, ES), passage (P; 3, 6, 9) and days (D; 3 vs. 5, 3 vs. 7, 5 vs. 7) in culture. Table S13: Analyses summary of eTC proliferation, metabolic activity, viability, SDS-PAGE densitometry and image intensity immunofluorescence for collagen type I, collagen type III, collagen type IV, collagen type V, collagen type VI, fibronectin, decorin and connexin-43in comparison to cells at passage 3, day 3, in foetal bovine serum (FBS) and without macromolecular crowding (MMC). Green background indicates significant increase, red background indicates significant decrease and white background indicates no significant difference. NS indicates not significant. SD indicates significant decrease. SI indicates significant increase. P indicates passage. D indicates day. ES indicates equine serum. Numbers indicate p values. Table S14: Collagen type I to collagen type III ratio as a function of time in culture (day 3, day 5, day 7), serum (foetal bovine serum, FBS; equine serum, ES), passage (3, 6, 9) and absence (−) or presence (+) of macromolecular crowding (MMC). Figure S1: Brightfield microscopy of eTCs as a function of serum, passage, MMC and time in culture. Figure S2: eTC cell number (proliferation) as a function of serum, passage, MMC and time in culture. * indicates significant (*p* < 0.05) increase in comparison to cells at passage 3, day 3, in FBS and without MMC. Figure S3: eTC metabolic activity as a function of serum, passage, MMC and time in culture. * indicates significant (*p* < 0.05) increase in comparison to cells at passage 3, day 3, in FBS and without MMC. # indicates significant (*p* < 0.05) decrease in comparison to cells at passage 3, day 3, in FBS and without MMC. Figure S4: eTC viability as a function of serum, passage, MMC and time in culture. * indicates significant (*p* < 0.05) increase in comparison to cells at passage 3, day 3, in FBS and without MMC. # indicates significant (*p* < 0.05) decrease in comparison to cells at passage 3, day 3, in FBS and without MMC.
