# Supplementary material for: Allogeneic Serum and Macromolecular Crowding Maintain Native Equine Tenocyte Function in Culture
Source: Cells. 2022 May 5;11(9):1562. doi: 10.3390/cells11091562 (PMC9103545; doi:10.3390/cells11091562)

Supplementary Information

Title

Allogeneic serum and macromolecular crowding maintain native equine tenocyte function in culture

Authors

Andrea Rampin (1, 2, 3), Ioannis Skoufos (1), Michael Raghunath (4), Athina Tzora (1), Nikolaos Diakakis (2), Nikitas Prassinos (2), Dimitrios I. Zeugolis* (3)

Affiliations

1. Laboratory of Animal Science, Nutrition and Biotechnology, School of Agriculture, University of Ioannina, Arta, Greece

2. School of Veterinary Medicine, Aristotle University of Thessaloniki, Thessaloniki, Greece

3. Regenerative, Modular & Developmental Engineering Laboratory (REMODEL), Charles Institute of Dermatology, Conway Institute of Biomolecular & Biomedical Research and School of Mechanical & Materials Engineering, University College Dublin (UCD), Dublin, Ireland

4. Center for Cell Biology and Tissue Engineering, Institute for Chemistry and Biotechnology, Zurich University of Applied Sciences, Wädenswil, Switzerland

* Corresponding Author: Dimitrios I. Zeugolis, REMODEL, UCD, Telephone: +353 17 16 18 84; Email: dimitrios.zevgolis@ucd.ie

Supplementary Table S1: eTC proliferation analysis as a function of macromolecular crowding (MMC), serum (foetal bovine serum, FBS; equine serum, ES), passage (P; 3, 6, 9) and days (D; 3 vs 5, 3 vs 7, 5 vs 7) in culture.

**MMC effect**				**Serum effect**				**Passage effect**					**Days in culture effect**				
**Passage**	**Day**	**Serum**	**P value**	**Passage**	**Day**	**±MMC**	**P value**	**Passage**	**Day**	**Serum**	**±MMC**	**P value**	**Day**	**Passage**	**Serum**	**±MMC**	**P value**
P3	D3	FBS	0.9595	P3	D3	-MMC	**0.026**	P3 Vs P6	D3	FBS	-MMC	0.1	D3 Vs D5	3	FBS	-MMC	**< 0.0001**
		ES	0.4770			+MMC	0.0835				+MMC	0.4847	D3 Vs D7				**< 0.0001**
	D5	FBS	0.1416		D5	-MMC	0.669			ES	-MMC	0.25	D5 Vs D7				0.2770
		ES	0.6108			+MMC	0.0659				+MMC	0.3123	D3 Vs D5			+MMC	**< 0.0001**
	D7	FBS	0.4268		D7	-MMC	0.2893		D5	FBS	-MMC	**< 0.0001**	D3 Vs D7				**< 0.0001**
		ES	0.5962			+MMC	**0.0002**				+MMC	**0.0417**	D5 Vs D7				**0.0267**
P6	D3	FBS	**0.0423**	P6	D3	-MMC	0.2164			ES	-MMC	0.21	D3 Vs D5		ES	-MMC	**0.0093**
		ES	**< 0.0001**			+MMC	0.1171				+MMC	**0.0001**	D3 Vs D7				**0.0053**
	D5	FBS	**0.0005**		D5	-MMC	**< 0.0001**		D7	FBS	-MMC	**< 0.0001**	D5 Vs D7				0.9273
		ES	**< 0.0001**			+MMC	**< 0.0001**				+MMC	**< 0.0001**	D3 Vs D5			+MMC	**0.0008**

	D7	FBS	0.2117		D7	-MMC	**< 0.0001**			ES	-MMC	**0.02**	D3 Vs D7				**0.0002**
		ES	**< 0.0001**			+MMC	**< 0.0001**				+MMC	**< 0.0001**	D5 Vs D7				0.6956
P9	D3	FBS	0.674	P9	D3	-MMC	**< 0.0001**	P3 Vs P9	D3	FBS	-MMC	**< 0.0001**	D3 Vs D5	6	FBS	-MMC	**< 0.0001**
		ES	**0.005**			+MMC	**< 0.0001**				+MMC	**< 0.0001**	D3 Vs D7				**< 0.0001**
	D5	FBS	0.537		D5	-MMC	**< 0.0001**			ES	-MMC	0.21	D5 Vs D7				0.1614
		ES	**< 0.0001**			+MMC	**< 0.0001**				+MMC	**0.0207**	D3 Vs D5			+MMC	**< 0.0001**
	D7	FBS	0.251		D7	-MMC	**< 0.0001**		D5	FBS	-MMC	**< 0.0001**	D3 Vs D7				**< 0.0001**
		ES	**0.001**			+MMC	**< 0.0001**				+MMC	**< 0.0001**	D5 Vs D7				0.1234
										ES	-MMC	**< 0.0001**	D3 Vs D5		ES	-MMC	**< 0.0001**
											+MMC	**< 0.0001**	D3 Vs D7				**< 0.0001**
									D7	FBS	-MMC	0.29	D5 Vs D7				0.1164
											+MMC	**< 0.0001**	D3 Vs D5			+MMC	**0.0073**
										ES	-MMC	**< 0.0001**	D3 Vs				**0.0002**

													D7				
											+MMC	**< 0.0001**	D5 Vs D7				0.7415
								P6 Vs P9	D3	FBS	-MMC	**< 0.0001**	D3 Vs D5	9	FBS	-MMC	**0.0019**
											+MMC	**< 0.0001**	D3 Vs D7				0.2586
										ES	-MMC	**0.01**	D5 Vs D7				**0.0022**
											+MMC	**< 0.0001**	D3 Vs D5			+MMC	**< 0.0001**
									D5	FBS	-MMC	0.35	D3 Vs D7				**0.0003**
											+MMC	**0.0055**	D5 Vs D7				**0.0279**
										ES	-MMC	**< 0.0001**	D3 Vs D5		ES	-MMC	**< 0.0001**
											+MMC	**0.0225**	D3 Vs D7				**< 0.0001**
									D7	FBS	-MMC	**0.03**	D5 Vs D7				0.9382
											+MMC	0.8858	D3 Vs D5			+MMC	**< 0.0001**
										ES	-MMC	**< 0.0001**	D3 Vs D7				**< 0.0001**

											+MMC	**0.0002**	D5 Vs D7				0.4409

Supplementary Table S2: eTC metabolic activity analysis as a function of macromolecular crowding (MMC), serum (foetal bovine serum, FBS; equine serum, ES), passage (P; 3, 6, 9) and days (D; 3 vs 5, 3 vs 7, 5 vs 7) in culture.

MMC effect				Serum effect				Passage effect					Days in culture effect				
**Passage**	**Day**	**Serum**	**P value**	**Passage**	**Day**	**±MMC**	**P value**	**Passage**	**Day**	**Serum**	**±MMC**	**P value**	**Day**	**Passage**	**Serum**	**±MMC**	**P value**
P3	D3	FBS	**0.0107**	P3	D3	-MMC	0.6925	P3 Vs P6	D3	FBS	-MMC	**0.0109**	D3 Vs D5	P3	FBS	-MMC	**< 0.0001**
		ES	**0.0007**			+MMC	0.3271				+MMC	0.2705	D3 Vs D7				**0.0156**
	D5	FBS	0.0719		D5	-MMC	**0.0002**			ES	-MMC	0.0999	D5 Vs D7				**< 0.0001**
		ES	0.1561			+MMC	**< 0.0001**				+MMC	0.2363	D3 Vs D5			+MMC	0.6979
	D7	FBS	**0.0412**		D7	-MMC	**< 0.0001**		D5	FBS	-MMC	0.0609	D3 Vs D7				**0.0092**
		ES	0.0934			+MMC	**< 0.0001**				+MMC	**0.0012**	D5 Vs D7				**< 0.0001**
P6	D3	FBS	**< 0.0001**	P6	D3	-MMC	0.1192			3081157 77 ES	-MMC	**< 0.0001**	D3 Vs D5		ES	-MMC	0.0740
		ES	**0.0002**			+MMC	0.1214				+MMC	**< 0.0001**	D3 Vs D7				**< 0.0001**
	D5	FBS	**0.0014**		D5	-MMC	**< 0.0001**		D7	FBS	-MMC	**0.0025**	D5 Vs D7				< 0.0001
		ES	0.0513			+MMC	**< 0.0001**				+MMC	**0.0001**	D3 Vs			+MMC	**0.0007**

													D5				
	D7	FBS	0.8694		D7	-MMC	**< 0.0001**			ES	-MMC	**0.0134**	D3 Vs D7				**< 0.0001**
		ES	0.2513			+MMC	**< 0.0001**				+MMC	0.1166	D5 Vs D7				**< 0.0001**
P9	D3	FBS	**0.0001**	P9	D3	-MMC	**< 0.0001**	P3 Vs P9	D3	FBS	-MMC	**0.0084**	D3 Vs D5	P6	FBS	-MMC	**0.0004**
		ES	**0.0001**			+MMC	**0.0043**				+MMC	0.2209	D3 Vs D7				**< 0.0001**
	D5	FBS	0.1685		D5	-MMC	**< 0.0001**			ES	-MMC	**0.0173**	D5 Vs D7				**< 0.0001**
		ES	0.1452			+MMC	**< 0.0001**				+MMC	0.1068	D3 Vs D5			+MMC	**< 0.0001**
	D7	FBS	0.1780		D7	-MMC	**< 0.0001**		D5	FBS	-MMC	**0.0211**	D3 Vs D7				**< 0.0001**
		ES	0.3141			+MMC	**< 0.0001**				+MMC	0.5116	D5 Vs D7				**< 0.0001**
										ES	-MMC	**< 0.0001**	D3 Vs D5		ES	-MMC	**0.0013**
											+MMC	**< 0.0001**	D3 Vs D7				**< 0.0001**
									D7	FBS	-MMC	**0.0086**	D5 Vs D7				**< 0.0001**
											+MMC	**0.0015**	D3 Vs D5			+MMC	**0.0018**

										ES	-MMC	**< 0.0001**	D3 Vs D7				**< 0.0001**
											+MMC	**< 0.0001**	D5 Vs D7				**< 0.0001**
								P6 Vs P9	D3	FBS	-MMC	0.8814	D3 Vs D5	P9	FBS	-MMC	**0.0192**
											+MMC	0.7496	D3 Vs D7				**< 0.0001**
										ES	-MMC	0.3959	D5 Vs D7				**< 0.0001**
											+MMC	0.6222	D3 Vs D5			+MMC	**0.0392**
									D5	FBS	-MMC	0.2072	D3 Vs D7				**< 0.0001**
											+MMC	**0.0219**	D5 Vs D7				**< 0.0001**
										ES	-MMC	**< 0.0001**	D3 Vs D5		ES	-MMC	**< 0.0001**
											+MMC	**< 0.0001**	D3 Vs D7				**0.0003**
									D7	FBS	-MMC	0.5042	D5 Vs D7				**< 0.0001**
											+MMC	0.2285	D3 Vs D5			+MMC	**0.0289**
										ES	-MMC	**0.0012**	D3 Vs				**< 0.0001**

													D7				
											+MMC	**< 0.0001**	D5 Vs D7				**< 0.0001**

Supplementary Table S3: eTC viability analysis as a function of macromolecular crowding (MMC), serum (foetal bovine serum, FBS; equine serum, ES), passage (P; 3, 6, 9) and days (D; 3 vs 5, 3 vs 7, 5 vs 7) in culture.

MMC effect				Serum effect				Passage effect					Days in culture effect				
**Passage**	**Day**	**Serum**	**P value**	**Passage**	**Day**	**±MMC**	**P value**	**Passage**	**Day**	**Serum**	**±MMC**	**P value**	**Day**	**Passage**	**Serum**	**±MMC**	**P value**
P3	D3	FBS	0.081	P3	D3	-MMC	**0.0069**	P3 Vs P6	D3	FBS	-MMC	**0.0041**	D3 Vs D5	P3	FBS	-MMC	**< 0.0001**
		ES	**0.033**			+MMC	**0.0035**				+MMC	**0.0050**	D3 Vs D7				**0.0013**
	D5	FBS	0.055		D5	-MMC	**< 0.0001**			ES	-MMC	**0.0161**	D5 Vs D7				**0.0010**
		ES	0.088			+MMC	**0.0070**				+MMC	0.9513	D3 Vs D5			+MMC	**0.0132**
	D7	FBS	**0.008**		D7	-MMC	**0.0002**		D5	FBS	-MMC	**0.0008**	D3 Vs D7				0.1938
		ES	0.149			+MMC	**0.0286**				+MMC	**0.0074**	D5 Vs D7				**0.0339**
P6	D3	FBS	0.557	P6	D3	-MMC	0.4990			ES	-MMC	0.4600	D3 Vs D5		ES	-MMC	**0.0011**
		ES	0.34			+MMC	0.5031				+MMC	0.4984	D3 Vs D7				**0.0236**
	D5	FBS	0.091		D5	-MMC	0.1571		D7	FBS	-MMC	0.8320	D5 Vs D7				0.4677
		ES	0.434			+MMC	0.2883				+MMC	**0.0442**	D3 Vs			+MMC	0.5442

													D5				
	D7	FBS	0.173		D7	-MMC	0.1020			ES	-MMC	0.6057	D3 Vs D7				0.5168
		ES	0.587			+MMC	0.1603				+MMC	0.1128	D5 Vs D7				0.9697
P9	D3	FBS	0.305	P9	D3	-MMC	0.0806	P3 Vs P9	D3	FBS	-MMC	**0.0050**	D3 Vs D5	P6	FBS	-MMC	**0.0414**
		ES	**0.034**			+MMC	0.4207				+MMC	**0.0043**	D3 Vs D7				0.1047
	D5	FBS	0.247		D5	-MMC	0.0957			ES	-MMC	0.3821	D5 Vs D7				0.2648
		ES	0.402			+MMC	**0.0041**				+MMC	0.9405	D3 Vs D5			+MMC	0.7739
	D7	FBS	0.076		D7	-MMC	**0.0115**		D5	FBS	-MMC	0.5034	D3 Vs D7				0.4443
		ES	0.163			+MMC	**0.0164**				+MMC	**0.0424**	D5 Vs D7				0.2804
										ES	-MMC	0.2157	D3 Vs D5		ES	-MMC	0.4609
											+MMC	0.0861	D3 Vs D7				0.9264
									D7	FBS	-MMC	0.4473	D5 Vs D7				0.4679
											+MMC	0.9457	D3 Vs D5			+MMC	0.7728

										ES	-MMC	0.4641	D3 Vs D7				0.7942
											+MMC	**0.0345**	D5 Vs D7				0.2657
								P6 Vs P9	D3	FBS	-MMC	0.5932	D3 Vs D5	P9	FBS	-MMC	0.0912
											+MMC	0.9944	D3 Vs D7				**0.0124**
										ES	-MMC	**0.0228**	D5 Vs D7				0.9917
											+MMC	0.8771	D3 Vs D5			+MMC	**0.0036**
									D5	FBS	-MMC	0.2153	D3 Vs D7				**0.0069**
											+MMC	**0.0034**	D5 Vs D7				0.3165
										ES	-MMC	0.7022	D3 Vs D5		ES	-MMC	0.4869
											+MMC	0.1070	D3 Vs D7				0.0120
									D7	FBS	-MMC	0.8461	D5 Vs D7				0.2135
											+MMC	**0.0089**	D3 Vs D5			+MMC	0.2039
										ES	-MMC	0.8658	D3 Vs				0.0581

													D7				
											+MMC	0.0641	D5 Vs D7				0.2073

Supplementary Table S4: eTC SDS-PAGE densitometry analysis as a function of macromolecular crowding (MMC), serum (foetal bovine serum, FBS; equine serum, ES), passage (P; 3, 6, 9) and days (D; 3 vs 5, 3 vs 7, 5 vs 7) in culture.

**MMC effect**				**Serum effect**				**Passage effect**					**Days in culture effect**				
**Passage**	**Day**	**Serum**	**P value**	**Passage**	**Day**	**±MMC**	**P value**	**Passage**	**Day**	**Serum**	**±MMC**	**P value**	**Day**	**Passage**	**Serum**	**±MMC**	**P value**
P3	D3	FBS	**0.0147**	P3	D3	-MMC	0.0591	P3 Vs P6	D3	FBS	-MMC	0.8941	D3 Vs D5	P3	FBS	-MMC	**0.0467**
		ES	**0.0119**			+MMC	0.0544				+MMC	0.0758	D3 Vs D7				**0.0453**
	D5	FBS	**0.0075**		D5	-MMC	0.1722			ES	-MMC	0.5883	D5 Vs D7				0.8249
		ES	**0.0007**			+MMC	0.3386				+MMC	0.2415	D3 Vs D5			+MMC	0.0783
	D7	FBS	**0.0142**		D7	-MMC	0.5018		D5	FBS	-MMC	0.2211	D3 Vs D7				0.1488
		ES	**0.0327**			+MMC	0.2685				+MMC	0.2884	D5 Vs D7				0.6280
P6	D3	FBS	**0.0118**	P6	D3	-MMC	0.0797			ES	-MMC	0.8842	D3 Vs D5		ES	-MMC	0.3079
		ES	**0.0034**			+MMC	**0.0265**				+MMC	**0.0021**	D3 Vs D7				0.6986
	D5	FBS	**0.0035**		D5	-MMC	0.8199		D7	FBS	-MMC	0.6901	D5 Vs D7				0.4796
		ES	**0.0002**			+MMC	0.3408				+MMC	0.4535	D3 Vs D5			+MMC	0.6997

	D7	FBS	**0.0031**		D7	-MMC	0.7188			ES	-MMC	0.5158	D3 Vs D7				0.6704
		ES	**0.0105**			+MMC	0.1796				+MMC	0.1194	D5 Vs D7				0.8371
P9	D3	FBS	**< 0.0001**	P9	D3	-MMC	0.9589	P3 Vs P9	D3	FBS	-MMC	0.1173	D3 Vs D5	P6	FBS	-MMC	0.0607
		ES	0.1000			+MMC	0.9602				+MMC	0.1849	D3 Vs D7				**0.0204**
	D5	FBS	**< 0.0001**		D5	-MMC	**0.0418**			ES	-MMC	0.2029	D5 Vs D7				0.3025
		ES	0.2000			+MMC	0.5751				+MMC	0.5019	D3 Vs D5			+MMC	0.0309
	D7	FBS	**0.0022**		D7	-MMC	0.2726		D5	FBS	-MMC	0.2156	D3 Vs D7				**0.0202**
		ES	**0.0176**			+MMC	0.2227				+MMC	0.1028	D5 Vs D7				0.1557
										ES	-MMC	0.0658	D3 Vs D5		ES	-MMC	0.7248
											+MMC	0.2119	D3 Vs D7				0.4836
									D7	FBS	-MMC	0.3386	D5 Vs D7				0.2141
											+MMC	0.7862	D3 Vs D5			+MMC	0.1214
										ES	-MMC	0.5412	D3 Vs				0.4665

													D7				
											+MMC	0.1002	D5 Vs D7				0.8182
								P6 Vs P9	D3	FBS	-MMC	0.0588	D3 Vs D5	P9	FBS	-MMC	0.3732
											+MMC	**0.0310**	D3 Vs D7				0.1293
										ES	-MMC	0.6846	D5 Vs D7				0.4441
											+MMC	0.7696	D3 Vs D5			+MMC	0.7583
									D5	FBS	-MMC	0.6428	D3 Vs D7				0.1009
											+MMC	0.6840	D5 Vs D7				0.0987
										ES	-MMC	0.0859	D3 Vs D5		ES	-MMC	0.0658
											+MMC	0.7033	D3 Vs D7				0.1134
									D7	FBS	-MMC	0.6855	D5 Vs D7				0.0546
											+MMC	0.4579	D3 Vs D5			+MMC	0.7087
										ES	-MMC	0.1464	D3 Vs D7				0.7675

											+MMC	0.6779	D5 Vs D7				0.8676

Supplementary Table S5: eTC collagen type I immunofluorescence image intensity analysis as a function of macromolecular crowding (MMC), serum (foetal bovine serum, FBS; equine serum, ES), passage (P; 3, 6, 9) and days (D; 3 vs 5, 3 vs 7, 5 vs 7) in culture.

**MMC effect**				**Serum effect**				**Passage effect**					**Days in culture effect**				
**Passage**	**Day**	**Serum**	**P value**	**Passage**	**Day**	**±MMC**	**P value**	**Passage**	**Day**	**Serum**	**±MMC**	**P value**	**Day**	**Passage**	**Serum**	**±MMC**	**P value**
P3	D3	FBS	**0.0061**	P3	D3	-MMC	0.1095	P3 Vs P6	D3	FBS	-MMC	0.1017	D3 Vs D5	P3	FBS	-MMC	0.6094
		ES	**0.0006**			+MMC	**0.0363**				+MMC	0.2471	D3 Vs D7				0.1826
	D5	FBS	**0.0014**		D5	-MMC	**0.0157**			ES	-MMC	0.0542	D5 Vs D7				**0.0140**
		ES	**0.0069**			+MMC	**0.0073**				+MMC	0.2760	D3 Vs D5			+MMC	0.2207
	D7	FBS	**0.0039**		D7	-MMC	**0.0004**		D5	FBS	-MMC	0.4504	D3 Vs D7				0.1604
		ES	**< 0.0001**			+MMC	**0.0071**				+MMC	0.2153	D5 Vs D7				0.6670
P6	D3	FBS	**< 0.0001**	P6	D3	-MMC	**0.0488**			ES	-MMC	0.7941	D3 Vs D5		ES	-MMC	**0.0243**
		ES	**0.0003**			+MMC	**0.0010**				+MMC	0.9805	D3 Vs D7				0.2060
	D5	FBS	**< 0.0001**		D5	-MMC	0.1418		D7	FBS	-MMC	**0.0138**	D5 Vs D7				0.6832
		ES	**0.0064**			+MMC	**0.0005**				+MMC	0.1848	D3 Vs D5			+MMC	**0.0293**

	D7	FBS	**0.0010**		D7	-MMC	0.0559			ES	-MMC	0.1073	D3 Vs D7				**0.0049**
		ES	**0.0182**			+MMC	0.0598				+MMC	0.2649	D5 Vs D7				0.4514
P9	D3	FBS	**0.0081**	P9	D3	-MMC	**0.0075**	P3 Vs P9	D3	FBS	-MMC	0.1300	D3 Vs D5	P6	FBS	-MMC	0.2608
		ES	**< 0.0001**			+MMC	0.7055				+MMC	0.1494	D3 Vs D7				**0.0058**
	D5	FBS	**0.0006**		D5	-MMC	**0.0039**			ES	-MMC	**0.0041**	D5 Vs D7				0.2246
		ES	**0.0013**			+MMC	**0.0047**				+MMC	**0.0133**	D3 Vs D5			+MMC	0.1377
	D7	FBS	**0.0001**		D7	-MMC	**0.0259**		D5	FBS	-MMC	**0.0469**	D3 Vs D7				0.1604
		ES	**0.0003**			+MMC	**0.0108**				+MMC	**0.0193**	D5 Vs D7				**0.0044**
										ES	-MMC	**0.0023**	D3 Vs D5		ES	-MMC	**0.0211**
											+MMC	**0.0027**	D3 Vs D7				0.3833
									D7	FBS	-MMC	**0.0003**	D5 Vs D7				0.0638
											+MMC	0.1474	D3 Vs D5			+MMC	0.0512
										ES	-MMC	**0.0140**	D3 Vs				0.2377

													D7				
											+MMC	**0.0007**	D5 Vs D7				0.5990
								P6 Vs P9	D3	FBS	-MMC	0.4195	D3 Vs D5	P9	FBS	-MMC	0.9021
											+MMC	0.3385	D3 Vs D7				0.6656
										ES	-MMC	0.1005	D5 Vs D7				0.4696
											+MMC	**0.0021**	D3 Vs D5			+MMC	0.1262
									D5	FBS	-MMC	0.3539	D3 Vs D7				0.3613
											+MMC	**0.0007**	D5 Vs D7				0.0691
										ES	-MMC	**0.0023**	D3 Vs D5		ES	-MMC	0.0705
											+MMC	**0.0027**	D3 Vs D7				0.7031
									D7	FBS	-MMC	**0.0086**	D5 Vs D7				0.0639
											+MMC	0.9088	D3 Vs D5			+MMC	**0.0311**
										ES	-MMC	0.0852	D3 Vs D7				0.1316

											+MMC	**0.0045**	D5 Vs D7				0.1204

Supplementary Table S6: eTC collagen type III immunofluorescence image intensity analysis as a function of macromolecular crowding (MMC), serum (foetal bovine serum, FBS; equine serum, ES), passage (P; 3, 6, 9) and days (D; 3 vs 5, 3 vs 7, 5 vs 7) in culture.

**MMC effect**				**Serum effect**				**Passage effect**					**Days in culture effect**				
**Passage**	**Day**	**Serum**	**P value**	**Passage**	**Day**	**±MMC**	**P value**	**Passage**	**Day**	**Serum**	**±MMC**	**P value**	**Day**	**Passage**	**Serum**	**±MMC**	**P value**
P3	D3	FBS	**0.0117**	P3	D3	-MMC	**0.0237**	P3 Vs P6	D3	FBS	-MMC	0.1120	D3 Vs D5	P3	FBS	-MMC	0.0626
		ES	**0.0082**			+MMC	**0.0095**				+MMC	0.7641	D3 Vs D7				0.8291
	D5	FBS	**0.0016**		D5	-MMC	**< 0.0001**			ES	-MMC	0.0665	D5 Vs D7				**0.0388**
		ES	**0.0012**			+MMC	**< 0.0001**				+MMC	0.7141	D3 Vs D5			+MMC	**0.0034**
	D7	FBS	0.1242		D7	-MMC	**0.0008**		D5	FBS	-MMC	**0.0024**	D3 Vs D7				**0.0054**
		ES	0.1469			+MMC	**0.0014**				+MMC	**0.0070**	D5 Vs D7				0.0757
P6	D3	FBS	0.3562	P6	D3	-MMC	0.0692			ES	-MMC	**0.0077**	D3 Vs D5		ES	-MMC	**0.0019**
		ES	**0.0487**			+MMC	**0.0470**				+MMC	**< 0.0001**	D3 Vs D7				**0.0013**
	D5	FBS	**0.0396**		D5	-MMC	**0.0287**		D7	FBS	-MMC	**0.0002**	D5 Vs D7				**0.0149**
		ES	**0.0030**			+MMC	**0.0349**				+MMC	**0.0011**	D3 Vs D5			+MMC	0.0776

	D7	FBS	**0.0066**		D7	-MMC	**0.0009**			ES	-MMC	**0.0009**	D3 Vs D7				**0.0329**
		ES	0.5106			+MMC	0.2444				+MMC	**0.0002**	D5 Vs D7				**0.0286**
P9	D3	FBS	**0.0018**	P9	D3	-MMC	**0.0022**	P3 Vs P9	D3	FBS	-MMC	0.5747	D3 Vs D5	P6	FBS	-MMC	**0.0153**
		ES	**0.0005**			+MMC	**0.0007**				+MMC	**0.0136**	D3 Vs D7				**0.0004**
	D5	FBS	**0.0015**		D5	-MMC	**0.0006**			ES	-MMC	**0.0130**	D5 Vs D7				0.1726
		ES	**0.0324**			+MMC	**0.0004**				+MMC	**0.0176**	D3 Vs D5			+MMC	**0.0143**
	D7	FBS	0.3828		D7	-MMC	**0.0138**		D5	FBS	-MMC	**0.0031**	D3 Vs D7				**0.0023**
		ES	0.6785			+MMC	**< 0.0001**				+MMC	**0.0027**	D5 Vs D7				0.3238
										ES	-MMC	**0.0020**	D3 Vs D5		ES	-MMC	**0.0052**
											+MMC	**0.0008**	D3 Vs D7				**0.0003**
									D7	FBS	-MMC	0.1272	D5 Vs D7				**0.0171**
											+MMC	**0.0001**	D3 Vs D5			+MMC	**0.0189**
										ES	-MMC	**0.0124**	D3 Vs				**0.0023**

													D7				
											+MMC	**< 0.0001**	D5 Vs D7				0.2341
								P6 Vs P9	D3	FBS	-MMC	**0.0497**	D3 Vs D5	P9	FBS	-MMC	0.3433
											+MMC	0.1317	D3 Vs D7				0.0990
										ES	-MMC	0.3888	D5 Vs D7				0.1190
											+MMC	**0.0206**	D3 Vs D5			+MMC	0.3372
									D5	FBS	-MMC	**0.0050**	D3 Vs D7				**0.0372**
											+MMC	**0.0181**	D5 Vs D7				**0.0087**
										ES	-MMC	0.1498	D3 Vs D5		ES	-MMC	**0.0017**
											+MMC	0.8441	D3 Vs D7				**0.0053**
									D7	FBS	-MMC	0.2445	D5 Vs D7				0.3186
											+MMC	0.6879	D3 Vs D5			+MMC	0.6659
										ES	-MMC	0.9179	D3 Vs D7				0.1493

											+MMC	0.8333	D5 Vs D7				0.2410

Supplementary Table S7: eTC collagen type IV immunofluorescence image intensity analysis as a function of macromolecular crowding (MMC), serum (foetal bovine serum, FBS; equine serum, ES), passage (P; 3, 6, 9) and days (D; 3 vs 5, 3 vs 7, 5 vs 7) in culture.

MMC effect				Serum effect				Passage effect					Days in culture effect				
**Passage**	**Day**	**Serum**	**P value**	**Passage**	**Day**	**±MMC**	**P value**	**Passage**	**Day**	**Serum**	**±MMC**	**P value**	**Day**	**Passage**	**Serum**	**±MMC**	**P value**
P3	D3	FBS	0.2150	P3	D3	-MMC	**< 0.0001**	P3 Vs P6	D3	FBS	-MMC	**< 0.0001**	D3 Vs D5	P3	FBS	-MMC	**< 0.0001**
		ES	0.1158			+MMC	**< 0.0001**				+MMC	**< 0.0001**	D3 Vs D7				**0.0002**
	D5	FBS	**0.0434**		D5	-MMC	**0.0052**			ES	-MMC	**0.0254**	D5 Vs D7				**0.0196**
		ES	0.2958			+MMC	0.3143				+MMC	0.0650	D3 Vs D5			+MMC	**< 0.0001**
	D7	FBS	0.0745		D7	-MMC	**0.0066**		D5	FBS	-MMC	**0.0019**	D3 Vs D7				**0.0003**
		ES	0.8850			+MMC	**0.0014**				+MMC	**0.0125**	D5 Vs D7				**0.0190**
P6	D3	FBS	**0.0004**	P6	D3	-MMC	0.1064			ES	-MMC	**0.0022**	D3 Vs D5		ES	-MMC	**0.0010**
		ES	**0.0250**			+MMC	0.2153				+MMC	**0.0002**	D3 Vs D7				**0.0022**
	D5	FBS	**0.0396**		D5	-MMC	**0.0049**		D7	FBS	-MMC	**0.0006**	D5 Vs D7				**0.0044**
		ES	**0.0129**			+MMC	**0.0029**				+MMC	0.1112	D3 Vs D5			+MMC	0.0733

	D7	FBS	0.4618		D7	-MMC	**0.0080**			ES	-MMC	0.0781	D3 Vs D7				**0.0002**
		ES	0.5820			+MMC	0.1858				+MMC	0.0705	D5 Vs D7				**0.0004**
	D3	FBS	**0.0085**		D3	-MMC	0.1206			FBS	-MMC	**< 0.0001**	D3 Vs D5				**0.0005**
		ES	**0.0014**			+MMC	**0.0040**		D3		+MMC	**0.0002**	D3 Vs D7			-MMC	**< 0.0001**
P9	D5	FBS	**0.0023**	P9	D5	-MMC	**0.0197**			ES	-MMC	0.2442	D5 Vs D7		FBS		**0.0029**
		ES	**0.0364**			+MMC	**< 0.0001**				+MMC	**0.0016**	D3 Vs D5				**0.0133**
	D7	FBS	0.0544		D7	-MMC	**0.0004**			FBS	-MMC	**0.0003**	D3 Vs D7			+MMC	**0.0642**
		ES	0.5374			+MMC	**0.0028**	P3 Vs P9	D5		+MMC	**0.0210**	D5 Vs D7	P6			0.8025
										ES	-MMC	0.1237	D3 Vs D5				**0.0010**
											+MMC	**0.0002**	D3 Vs D7			-MMC	**0.0004**
										FBS	-MMC	**0.0074**	D5 Vs D7		ES		0.8377
									D7		+MMC	0.0902	D3 Vs D5			+MMC	**0.0011**
										ES	-MMC	0.2311	D3 Vs				**0.0190**

													D7				
											+MMC	**0.0402**	D5 Vs D7				0.0574
								P6 Vs P9	D3	FBS	-MMC	**0.0148**	D3 Vs D5	P9	FBS	-MMC	0.1230
											+MMC	0.1403	D3 Vs D7				**0.0467**
										ES	-MMC	0.1493	D5 Vs D7				0.1470
											+MMC	0.2434	D3 Vs D5			+MMC	0.2820
									D5	FBS	-MMC	**0.0004**	D3 Vs D7				0.4185
											+MMC	**0.0065**	D5 Vs D7				0.1291
										ES	-MMC	**0.0132**	D3 Vs D5		ES	-MMC	**0.0270**
											+MMC	**0.0011**	D3 Vs D7				**0.0003**
									D7	FBS	-MMC	**< 0.0001**	D5 Vs D7				0.1089
											+MMC	0.0642	D3 Vs D5			+MMC	0.0971
										ES	-MMC	**0.0118**	D3 Vs D7				0.8480

											+MMC	**0.0273**	D5 Vs<br>D7				**0.0349**

Supplementary Table S8: eTC collagen type V immunofluorescence image intensity analysis as a function of macromolecular crowding (MMC), serum (foetal bovine serum, FBS; equine serum, ES), passage (P; 3, 6, 9) and days (D; 3 vs 5, 3 vs 7, 5 vs 7) in culture.

**MMC effect**				**Serum effect**				**Passage effect**					**Days in culture effect**				
**Passage**	**Day**	**Serum**	**P value**	**Passage**	**Day**	**±MMC**	**P value**	**Passage**	**Day**	**Serum**	**±MMC**	**P value**	**Day**	**Passage**	**Serum**	**±MMC**	**P value**
P3	D3	FBS	**0.0001**	P3	D3	-MMC	**0.0011**	P3 Vs P6	D3	FBS	-MMC	0.5644	D3 Vs D5	P3	FBS	-MMC	**0.0049**
		ES	**< 0.0001**			+MMC	**0.0009**				+MMC	**0.0081**	D3 Vs D7				**0.0220**
	D5	FBS	**0.0003**		D5	-MMC	**0.0070**			ES	-MMC	**0.0215**	D5 Vs D7				0.0870
		ES	**0.0004**			+MMC	**0.0289**				+MMC	**< 0.0001**	D3 Vs D5			+MMC	**0.0001**
	D7	FBS	**0.0032**		D7	-MMC	**0.0071**		D5	FBS	-MMC	**0.0026**	D3 Vs D7				**0.0012**
		ES	**0.0110**			+MMC	0.7102				+MMC	**< 0.0001**	D5 Vs D7				0.0829
P6	D3	FBS	**0.0037**	P6	D3	-MMC	0.1041			ES	-MMC	**0.0014**	D3 Vs D5		ES	-MMC	**0.0027**
		ES	**< 0.0001**			+MMC	**0.0383**				+MMC	**0.0009**	D3 Vs D7				0.8691
	D5	FBS	**0.0002**		D5	-MMC	**0.0230**		D7	FBS	-MMC	**0.0007**	D5 Vs D7				**0.0011**
		ES	**0.0010**			+MMC	0.6497				+MMC	0.0516	D3 Vs D5			+MMC	0.8579

	D7	FBS	**0.0001**		D7	-MMC	**0.0229**			ES	-MMC	**< 0.0001**	D3 Vs D7				0.3284
		ES	**0.0001**			+MMC	0.1703				+MMC	**0.0001**	D5 Vs D7				0.4098
P9	D3	FBS	**0.0004**	P9	D3	-MMC	**0.0198**	P3 Vs P9	D3	FBS	-MMC	0.4979	D3 Vs D5	P6	FBS	-MMC	**0.0061**
		ES	**0.0002**			+MMC	**0.0149**				+MMC	**0.0009**	D3 Vs D7				**0.0208**
	D5	FBS	**< 0.0001**		D5	-MMC	**0.0311**			ES	-MMC	**0.0002**	D5 Vs D7				0.2479
		ES	**0.0009**			+MMC	**0.0077**				+MMC	**0.0003**	D3 Vs D5			+MMC	**0.0498**
	D7	FBS	**0.0010**		D7	-MMC	0.2230		D5	FBS	-MMC	0.4030	D3 Vs D7				**0.0266**
		ES	**< 0.0001**			+MMC	**0.0015**				+MMC	**< 0.0001**	D5 Vs D7				0.3214
										ES	-MMC	**0.0044**	D3 Vs D5		ES	-MMC	**0.0056**
											+MMC	**0.0011**	D3 Vs D7				**0.0171**
									D7	FBS	-MMC	0.3909	D5 Vs D7				**0.0374**
											+MMC	**0.0019**	D3 Vs D5			+MMC	**0.0032**
										ES	-MMC	**0.0055**	D3 Vs				**0.0007**

													D7				
											+MMC	**< 0.0001**	D5 Vs D7				0.4237
								P6 Vs P9	D3	FBS	-MMC	0.3589	D3 Vs D5	P9	FBS	-MMC	0.0511
											+MMC	0.8596	D3 Vs D7				0.4908
										ES	-MMC	**0.0040**	D5 Vs D7				0.1832
											+MMC	**0.0800**	D3 Vs D5			+MMC	**0.0050**
									D5	FBS	-MMC	**0.0028**	D3 Vs D7				**0.0139**
											+MMC	**0.0002**	D5 Vs D7				0.5366
										ES	-MMC	**0.0322**	D3 Vs D5		ES	-MMC	0.2542
											+MMC	0.0962	D3 Vs D7				0.0751
									D7	FBS	-MMC	**0.0112**	D5 Vs D7				0.6350
											+MMC	**0.0005**	D3 Vs D5			+MMC	**0.0173**
										ES	-MMC	0.2489	D3 Vs D7				**0.0007**

											+MMC	**0.0492**	D5 Vs D7				0.9609

Supplementary Table S9: eTC collagen type VI immunofluorescence image intensity analysis as a function of macromolecular crowding (MMC), serum (foetal bovine serum, FBS; equine serum, ES), passage (P; 3, 6, 9) and days (D; 3 vs 5, 3 vs 7, 5 vs 7) in culture.

MMC effect				Serum effect				Passage effect					Days in culture effect				
**Passage**	**Day**	**Serum**	**P value**	**Passage**	**Day**	**±MMC**	**P value**	**Passage**	**Day**	**Serum**	**±MMC**	**P value**	**Day**	**Passage**	**Serum**	**±MMC**	**P value**
P3	D3	FBS	**0.0011**	P3	D3	-MMC	0.2168	P3 Vs P6	D3	FBS	-MMC	0.5189	D3 Vs D5	P3	FBS	-MMC	0.9411
		ES	**0.0418**			+MMC	0.1859				+MMC	**0.0435**	D3 Vs D7				**0.0006**
	D5	FBS	**0.0046**		D5	-MMC	0.0850			ES	-MMC	0.9609	D5 Vs D7				**0.0006**
		ES	**0.0120**			+MMC	0.4997				+MMC	**0.0440**	D3 Vs D5			+MMC	0.0553
	D7	FBS	**0.0066**		D7	-MMC	**0.0264**		D5	FBS	-MMC	0.1000	D3 Vs D7				< 0.0001
		ES	**0.0010**			+MMC	**0.0008**				+MMC	**< 0.0001**	D5 Vs D7				**< 0.0001**
P6	D3	FBS	0.1000	P6	D3	-MMC	0.1655			ES	-MMC	**< 0.0001**	D3 Vs D5		ES	-MMC	0.7548
		ES	**< 0.0001**			+MMC	0.1000				+MMC	**0.0363**	D3 Vs D7				**0.0002**
	D5	FBS	**0.0068**		D5	-MMC	0.1000		D7	FBS	-MMC	0.0502	D5 Vs D7				**0.0001**
		ES	0.1000			+MMC	0.0595				+MMC	0.6628	D3 Vs D5			+MMC	0.6026

	D7	FBS	0.6074		D7	-MMC	**0.0012**			ES	-MMC	0.2628	D3 Vs D7				**0.0009**
		ES	**0.0013**			+MMC	0.1594				+MMC	0.2818	D5 Vs D7				**0.0007**
P9	D3	FBS	**0.0165**	P9	D3	-MMC	0.2383	P3 Vs P9	D3	FBS	-MMC	0.1000	D3 Vs D5	P6	FBS	-MMC	**0.0021**
		ES	0.0784			+MMC	**0.0077**				+MMC	**0.0012**	D3 Vs D7				**0.0002**
	D5	FBS	**0.0020**		D5	-MMC	0.0619			ES	-MMC	0.0855	D5 Vs D7				**0.0396**
		ES	**0.0057**			+MMC	**0.0128**				+MMC	0.2929	D3 Vs D5			+MMC	0.2460
	D7	FBS	**0.0363**		D7	-MMC	0.9865		D5	FBS	-MMC	0.4628	D3 Vs D7				0.3899
		ES	**0.0242**			+MMC	0.6174				+MMC	0.0949	D5 Vs D7				0.5841
										ES	-MMC	0.1000	D3 Vs D5		ES	-MMC	**0.0003**
											+MMC	**0.0146**	D3 Vs D7				**< 0.0001**
									D7	FBS	-MMC	**0.0026**	D5 Vs D7				**0.0004**
											+MMC	**0.0010**	D3 Vs D5			+MMC	0.0821
										ES	-MMC	**0.0006**	D3 Vs				**0.0010**

													D7				
											+MMC	**0.0391**	D5 Vs D7				0.3937
								P6 Vs P9	D3	FBS	-MMC	0.0943	D3 Vs D5	P9	FBS	-MMC	0.0591
											+MMC	0.7000	D3 Vs D7				0.2280
										ES	-MMC	0.0811	D5 Vs D7				0.2288
											+MMC	0.2765	D3 Vs D5			+MMC	**0.0006**
									D5	FBS	-MMC	**0.0017**	D3 Vs D7				**0.0461**
											+MMC	**< 0.0001**	D5 Vs D7				0.1908
										ES	-MMC	0.1665	D3 Vs D5		ES	-MMC	0.6897
											+MMC	0.5939	D3 Vs D7				0.7978
									D7	FBS	-MMC	**0.0006**	D5 Vs D7				0.4406
											+MMC	**0.0054**	D3 Vs D5			+MMC	**0.0303**
										ES	-MMC	**0.0002**	D3 Vs D7				0.2240

											+MMC	**0.0274**	D5 Vs D7				0.3028

Supplementary Table S10: TC fibronectin immunofluorescence image intensity analysis as a function of macromolecular crowding (MMC), serum (foetal bovine serum, FBS; equine serum, ES), passage (P; 3, 6, 9) and days (D; 3 vs 5, 3 vs 7, 5 vs 7) in culture.

MMC effect				Serum effect				Passage effect					Days in culture effect				
**Passage**	**Day**	**Serum**	**P value**	**Passage**	**Day**	**±MMC**	**P value**	**Passage**	**Day**	**Serum**	**±MMC**	**P value**	**Day**	**Passage**	**Serum**	**±MMC**	**P value**
P3	D3	FBS	0.1000	P3	D3	-MMC	0.1000	P3 Vs P6	D3	FBS	-MMC	**0.0002**	D3 Vs D5	P3	FBS	-MMC	**< 0.0001**
		ES	0.1298			+MMC	**0.0127**				+MMC	**0.0218**	D3 Vs D7				**< 0.0001**
	D5	FBS	**0.0378**		D5	-MMC	**0.0301**			ES	-MMC	**0.0085**	D5 Vs D7				0.5176
		ES	0.2517			+MMC	**0.0005**				+MMC	**0.0092**	D3 Vs D5			+MMC	**0.0003**
	D7	FBS	**0.0155**		D7	-MMC	0.3337		D5	FBS	-MMC	0.7765	D3 Vs D7				**0.0002**
		ES	**0.0054**			+MMC	**0.0093**				+MMC	0.5047	D5 Vs D7				0.0942
P6	D3	FBS	0.4293	P6	D3	-MMC	0.4387			ES	-MMC	**0.0023**	D3 Vs D5		ES	-MMC	**0.0133**
		ES	**0.0044**			+MMC	**0.0216**				+MMC	**< 0.0001**	D3 Vs D7				**0.0323**
	D5	FBS	**0.0335**		D5	-MMC	0.7000		D7	FBS	-MMC	**0.0020**	D5 Vs D7				**0.0172**
		ES	0.1000			+MMC	**0.0013**				+MMC	**< 0.0001**	D3 Vs D5			+MMC	**0.0022**

	D7	FBS	**0.0038**		D7	-MMC	**0.0021**			ES	-MMC	0.1747	D3 Vs D7				**0.0041**
		ES	**< 0.0001**			+MMC	**< 0.0001**				+MMC	**< 0.0001**	D5 Vs D7				**0.0056**
	D3	FBS	0.1430		D3	-MMC	**< 0.0001**			FBS	-MMC	**< 0.0001**	D3 Vs D5				**0.0010**
		ES	**0.0025**			+MMC	**< 0.0001**		D3		+MMC	**0.0001**	D3 Vs D7			-MMC	**< 0.0001**
P9	D5	FBS	0.5781	P9	D5	-MMC	**< 0.0001**			ES	-MMC	**0.0030**	D5 Vs D7		FBS		0.0589
		ES	0.1000			+MMC	0.1000				+MMC	**0.0011**	D3 Vs D5				**0.0005**
	D7	FBS	0.0974		D7	-MMC	**0.0003**			FBS	-MMC	**0.0097**	D3 Vs D7			+MMC	**0.0002**
		ES	**0.0037**			+MMC	**< 0.0001**	P3 Vs P9			+MMC	**0.0002**	D5 Vs D7	P6			**< 0.0001**
									D5	ES	-MMC	**< 0.0001**	D3 Vs D5				**< 0.0001**
											+MMC	**0.0003**	D3 Vs D7			-MMC	**< 0.0001**
											-MMC	**0.0149**	D5 Vs D7		ES		0.4752
									D7	FBS	+MMC	**0.0006**	D3 Vs D5			+MMC	**0.0021**
										ES	-MMC	**0.0004**	D3 Vs				**0.0005**

													D7				
											+MMC	**0.0001**	D5 Vs D7				0.1026
								P3 Vs P6	D3	FBS	-MMC	**< 0.0001**	D3 Vs D5	P9	FBS	-MMC	0.1230
											+MMC	**0.0003**	D3 Vs D7				0.4940
										ES	-MMC	**0.0312**	D5 Vs D7				0.0983
											+MMC	**0.0046**	D3 Vs D5			+MMC	**0.0289**
									D5	FBS	-MMC	**0.0389**	D3 Vs D7				**0.0493**
											+MMC	**< 0.0001**	D5 Vs D7				0.7634
										ES	-MMC	**< 0.0001**	D3 Vs D5		ES	-MMC	**0.0001**
											+MMC	0.2158	D3 Vs D7				**0.0007**
									D7	FBS	-MMC	0.2721	D5 Vs D7				0.3313
											+MMC	0.1131	D3 Vs D5			+MMC	**0.0011**
										ES	-MMC	**0.0009**	D3 Vs D7				**0.0018**

											+MMC	**0.0027**	D5 Vs<br>D7				0.1276

Supplementary Table S11: TC decorin immunofluorescence image intensity analysis as a function of macromolecular crowding (MMC), serum (foetal bovine serum, FBS; equine serum, ES), passage (P; 3, 6, 9) and days (D; 3 vs 5, 3 vs 7, 5 vs 7) in culture.

**MMC effect**				**Serum effect**				**Passage effect**					**Days in culture effect**				
**Passage**	**Day**	**Serum**	**P value**	**Passage**	**Day**	**±MMC**	**P value**	**Passage**	**Day**	**Serum**	**±MMC**	**P value**	**Day**	**Passage**	**Serum**	**±MMC**	**P value**
P3	D3	FBS	0.6579	P3	D3	-MMC	0.0722	P3 Vs P6	D3	FBS	-MMC	**< 0.0001**	D3 Vs D5	P3	FBS	-MMC	**< 0.0001**
		ES	0.1409			+MMC	0.0765				+MMC	0.1841	D3 Vs D7				**< 0.0001**
	D5	FBS	0.7285		D5	-MMC	0.0867			ES	-MMC	0.0573	D5 Vs D7				**0.0218**
		ES	0.2248			+MMC	0.1000				+MMC	0.0722	D3 Vs D5			+MMC	0.0503
	D7	FBS	0.0950		D7	-MMC	0.1000		D5	FBS	-MMC	0.1244	D3 Vs D7				**0.0300**
		ES	0.7000			+MMC	**< 0.0001**				+MMC	0.2716	D5 Vs D7				**0.0056**
P6	D3	FBS	0.6531	P6	D3	-MMC	**0.0005**			ES	-MMC	0.3600	D3 Vs D5		ES	-MMC	**0.0401**
		ES	**0.0072**			+MMC	0.0722				+MMC	**0.0007**	D3 Vs D7				0.7433
	D5	FBS	**0.0327**		D5	-MMC	0.1814		D7	FBS	-MMC	0.3323	D5 Vs D7				0.1223
		ES	0.5446			+MMC	**0.0288**				+MMC	0.6651	D3 Vs D5			+MMC	0.2877

	D7	FBS	0.3727		D7	-MMC	**0.0336**			ES	-MMC	0.4561	D3 Vs D7				0.0541
		ES	0.9599			+MMC	**0.0056**				+MMC	0.2000	D5 Vs D7				**< 0.0001**
	D3	FBS	**0.0005**		D3	-MMC	**0.0008**			FBS	-MMC	0.0722	D3 Vs D5				**0.0059**
		ES	**0.0040**			+MMC	0.0765		D3		+MMC	0.0765	D3 Vs D7			-MMC	**< 0.0001**
P9	D5	FBS	**0.0021**	P9	D5	-MMC	0.0545			ES	-MMC	0.1393	D5 Vs D7		FBS		**0.0032**
		ES	0.4682			+MMC	**0.0401**				+MMC	0.6579	D3 Vs D5				**0.0275**
	D7	FBS	**0.0278**		D7	-MMC	0.5276			FBS	-MMC	**0.0190**	D3 Vs D7			+MMC	**0.0106**
		ES	0.6951			+MMC	**0.0018**	P3 Vs P9			+MMC	0.4304	D5 Vs D7	P6			**0.0001**
									D5	ES	-MMC	0.2291	D3 Vs D5				0.2186
											+MMC	**0.0156**	D3 Vs D7			-MMC	**0.0435**
										FBS	-MMC	0.0558	D5 Vs D7		ES		0.8229
									D7		+MMC	0.5267	D3 Vs D5			+MMC	**0.0143**
										ES	-MMC	0.1838	D3 Vs				0.6328

													D7				
											+MMC	**0.0004**	D5 Vs D7				0.0613
								P6 Vs P9	D3	FBS	-MMC	**0.0002**	D3 Vs D5	P9	FBS	-MMC	0.0833
											+MMC	0.0722	D3 Vs D7				0.0115
										ES	-MMC	**0.0045**	D5 Vs D7				**0.0055**
											+MMC	**< 0.0001**	D3 Vs D5			+MMC	**0.0034**
									D5	FBS	-MMC	0.2310	D3 Vs D7				**0.0006**
											+MMC	0.2070	D5 Vs D7				**< 0.0001**
										ES	-MMC	0.8611	D3 Vs D5		ES	-MMC	0.4260
											+MMC	**0.0131**	D3 Vs D7				0.4647
									D7	FBS	-MMC	**0.0411**	D5 Vs D7				0.3007
											+MMC	0.9181	D3 Vs D5			+MMC	**0.0077**
										ES	-MMC	0.3856	D3 Vs D7				0.0932

											+MMC	0.0674	D5 Vs<br>D7				**0.0394**

Supplementary Table S12: eTC connexin 43 immunofluorescence image intensity analysis as a function of macromolecular crowding (MMC), serum (foetal bovine serum, FBS; equine serum, ES), passage (P; 3, 6, 9) and days (D; 3 vs 5, 3 vs 7, 5 vs 7) in culture.

**MMC effect**				**Serum effect**				**Passage effect**					**Days in culture effect**				
**Passage**	**Day**	**Serum**	**P value**	**Passage**	**Day**	**±MMC**	**P value**	**Passage**	**Day**	**Serum**	**±MMC**	**P value**	**Day**	**Passage**	**Serum**	**±MMC**	**P value**
P3	D3	FBS	0.2856	P3	D3	-MMC	**0.0062**	P3 Vs P6	D3	FBS	-MMC	0.1000	D3 Vs D5	P3	FBS	-MMC	**0.0497**
		ES	0.3551			+MMC	0.1000				+MMC	0.0765	D3 Vs D7				**0.0061**
	D5	FBS	0.1000		D5	-MMC	**0.0004**			ES	-MMC	0.2250	D5 Vs D7				**0.0004**
		ES	0.6697			+MMC	0.1000				+MMC	0.3491	D3 Vs D5			+MMC	0.2758
	D7	FBS	**0.0360**		D7	-MMC	**0.0318**		D5	FBS	-MMC	0.1000	D3 Vs D7				0.0607
		ES	0.2679			+MMC	0.1000				+MMC	0.1000	D5 Vs D7				**0.0010**
P6	D3	FBS	0.0800	P6	D3	-MMC	0.1853			ES	-MMC	0.4574	D3 Vs D5		ES	-MMC	0.6756
		ES	0.9088			+MMC	**0.0458**				+MMC	**0.0171**	D3 Vs D7				**0.0322**
	D5	FBS	0.2074		D5	-MMC	0.4280		D7	FBS	-MMC	0.1000	D5 Vs D7				**0.0331**
		ES	0.7000			+MMC	0.1430				+MMC	**< 0.0001**	D3 Vs D5			+MMC	0.0828

	D7	FBS	0.7295		D7	-MMC	0.0769			ES	-MMC	0.1000	D3 Vs D7				**0.0016**
		ES	0.2634			+MMC	**0.0140**				+MMC	**0.0013**	D5 Vs D7				**0.0032**
	D3	FBS	0.2626		D3	-MMC	0.1000			FBS	-MMC	0.1000	D3 Vs D5				0.2770
		ES	0.1000			+MMC	0.4977		D3		+MMC	0.1000	D3 Vs D7			-MMC	**0.0203**
P9	D5	FBS	0.1003	P9	D5	-MMC	0.6467			ES	-MMC	0.1156	D5 Vs D7		FBS		0.0916
		ES	0.7798			+MMC	0.2653				+MMC	0.3080	D3 Vs D5				**0.0430**
	D7	FBS	0.2000		D7	-MMC	0.1000			FBS	-MMC	**0.0006**	D3 Vs D7			+MMC	**0.0395**
		ES	0.4247			+MMC	0.1314	P3 Vs P9	D5		+MMC	0.1000	D5 Vs D7	P6			0.3180
										ES	-MMC	0.8897	D3 Vs D5				0.3249
											+MMC	0.4834	D3 Vs D7			-MMC	0.7117
										FBS	-MMC	0.1000	D5 Vs D7		ES		0.3726
									D7		+MMC	**< 0.0001**	D3 Vs D5			+MMC	0.5040
										ES	-MMC	0.1000	D3 Vs				0.0581

													D7				
											+MMC	**0.0014**	D5 Vs D7				0.0802
								P6 Vs P9	D3	FBS	-MMC	0.0642	D3 Vs D5	P9	FBS	-MMC	**0.0408**
											+MMC	0.1000	D3 Vs D7				0.0536
										ES	-MMC	0.0765	D5 Vs D7				**0.0300**
											+MMC	0.8411	D3 Vs D5			+MMC	**0.0304**
									D5	FBS	-MMC	0.0570	D3 Vs D7				0.3726
											+MMC	**0.0379**	D5 Vs D7				**0.0260**
										ES	-MMC	0.6284	D3 Vs D5		ES	-MMC	0.2328
											+MMC	0.7000	D3 Vs D7				**0.0015**
									D7	FBS	-MMC	0.7000	D5 Vs D7				0.3159
											+MMC	0.8473	D3 Vs D5			+MMC	0.1311
										ES	-MMC	0.6031	D3 Vs D7				0.2754

											+MMC	0.6295	D5 Vs D7				0.1826

Supplementary Table S13: Analyses summary of eTC proliferation, metabolic activity, viability, SDS-PAGE densitometry and image intensity immunofluorescence for collagen type I, collagen type III, collagen type IV, collagen type V, collagen type VI, fibronectin, decorin and connexin-43in comparison to cells at passage 3, day 3, in foetal bovine serum (FBS) and without macromolecular crowding (MMC). Green background indicates significant increase, red background indicates significant decrease and white background indicates no significant difference. NS indicates not significant. SD indicates significant decrease. SI indicates significant increase. P indicates passage. D indicates day. ES indicates equine serum. Numbers indicate p values.

	**Proliferation**	**Metabolic activity**	**Viability**	**SDS-PAGE**	**Collagen I**	**Collagen III**	**Collagen IV**	**Collagen V**	**Collagen VI**	**Fibronectin**	**Decorin**	**Connexin 43**	**Summary**
P3 D3 FBS +MMC	0.96	**0.0107**	**0.0299**	**0.0147**	**0.0061**	**0.0117**	0.2150	**0.0001**	**0.0011**	**0.0034**	0.6579	0.2856	4 NS 2 SD 6 SI
P3 D3 ES -MMC	**0.026**	0.6925	**0.0069**	0.0591	0.1095	**0.0237**	**< 0.0001**	**0.0011**	0.2168	**0.0003**	0.0722	**0.0062**	5 NS 4 SD 3 SI
P3 D3 ES +MMC	0.1048	**0.0003**	**0.0031**	0.0708	**0.0017**	**0.0054**	**< 0.0001**	**0.0019**	0.1000	0.1000	0.0722	0.1000	6 NS 2 SD 4 SI
P3 D5 FBS -MMC	**< 0.0001**	**< 0.0001**	**< 0.0001**	**0.0467**	0.6094	0.0626	**< 0.0001**	**0.0049**	0.9411	**< 0.0001**	0.0765	**0.0497**	4 NS 6 SD 2 SI
P3 D5 FBS	**< 0.0001**	**< 0.0001**	**0.0319**	**0.0410**	**0.0018**	0.3338	**< 0.0001**	**0.0213**	**0.0041**	**< 0.0001**	0.0765	**0.0077**	2 NS 4 SD

+MMC													6 SI
P3 D5<br>ES<br>-MMC	**< 0.0001**	**0.0138**	**0.0032**	0.1170	0.0674	**0.0015**	**< 0.0001**	**0.0004**	0.0639	**< 0.0001**	**< 0.0001**	0.1000	4 NS<br>5 SD<br>3 SI
P3 D5<br>ES<br>+MMC	**< 0.0001**	**0.0467**	**0.0025**	**0.0226**	**0.0331**	**0.0001**	**< 0.0001**	**0.0072**	**0.0187**	**< 0.0001**	**< 0.0001**	0.1000	1 NS<br>4 SD<br>7 SI
P3 D7<br>FBS<br>-MMC	**< 0.0001**	**0.0156**	**0.0013**	**0.0453**	0.1826	0.8291	**0.0002**	**0.0220**	0.1000	**< 0.0001**	**< 0.0001**	0.1000	4 NS<br>6 SD<br>2 SI
P3 D7<br>FBS<br>+MMC	**< 0.0001**	0.8006	0.7727	**0.0481**	**0.0033**	0.0996	**0.0003**	**0.0153**	**< 0.0001**	**< 0.0001**	**< 0.0001**	0.1000	4 NS<br>3 SD<br>5 SI
P3 D7<br>ES<br>-MMC	**< 0.0001**	**< 0.0001**	**0.0040**	0.0734	0.0794	**0.0008**	0.1493	**0.0011**	**0.0002**	**< 0.0001**	0.0765	0.1509	5 NS<br>2 SD<br>5 SI
P3 D7<br>ES<br>+MMC	**< 0.0001**	**< 0.0001**	**0.0025**	0.1658	**0.0093**	**0.0016**	**0.0323**	0.1132	**< 0.0001**	**< 0.0001**	**< 0.0001**	**0.0250**	2 NS<br>1 SD<br>9 SI
P6 D3<br>FBS<br>-MMC	**< 0.0001**	**0.0109**	**0.0041**	0.8941	0.1017	0.1120	**< 0.0001**	0.5644	0.5189	**0.0002**	**< 0.0001**	0.1000	6 NS<br>4 SD<br>2 SI
P6 D3<br>FBS<br>+MMC	**0.0396**	**< 0.0001**	**0.0036**	**0.0121**	**< 0.0001**	0.0917	**< 0.0001**	**0.0035**	0.1000	0.7000	0.0722	**0.0136**	4 NS<br>3 SD<br>5 SI

P6 D3<br>ES<br>-MMC	0.5498	**0.0295**	**0.0036**	0.1198	0.3948	**0.0109**	**< 0.0001**	0.0581	0.1643	**< 0.0001**	0.0722	0.0765	7 NS<br>3 SD<br>2 SI
P6 D3<br>ES<br>+MMC	0.0836	**< 0.0001**	**0.0030**	**0.0120**	**0.0011**	**0.0201**	0.1496	**< 0.0001**	**< 0.0001**	**0.0019**	0.0722	0.1000	4 NS<br>2 SD<br>6 SI
P6 D5<br>FBS<br>-MMC	0.174	**< 0.0001**	0.4811	0.1113	0.3466	**0.0063**	**0.0013**	**0.0045**	0.1000	0.1000	0.0765	0.1000	8NS<br>2 SD<br>2 SI
P6 D5<br>FBS<br>+MMC	**< 0.0001**	**0.0003**	**0.0032**	**0.0111**	**< 0.0001**	**0.0064**	0.3848	**< 0.0001**	**< 0.0001**	**< 0.0001**	**< 0.0001**	0.1000	2 NS<br>3 SD<br>7 SI
P6 D5<br>ES<br>-MMC	**< 0.0001**	**< 0.0001**	0.1920	0.1488	0.0701	**0.0019**	**0.0187**	**0.0127**	**0.0001**	**< 0.0001**	0.0765	**0.0058**	4NS<br>3 SD<br>5SI
P6 D5<br>ES<br>+MMC	**< 0.0001**	**< 0.0001**	**0.0026**	**0.0010**	**0.0309**	**< 0.0001**	**0.0007**	**0.0008**	0.1000	**< 0.0001**	**< 0.0001**	0.1000	2 NS<br>3 SD<br>7 SI
P6 D7<br>FBS<br>-MMC	**< 0.0001**	**0.0004**	0.1986	0.0529	0.8434	**0.0002**	0.9766	**0.0145**	0.1000	**< 0.0001**	**< 0.0001**	0.1000	6 NS<br>2 SD<br>4 SI
P6 D7<br>FBS<br>+MMC	**0.0091**	**< 0.0001**	**0.0044**	**0.0288**	**0.0015**	**0.0010**	0.4613	**< 0.0001**	0.1000	**< 0.0001**	**< 0.0001**	0.1000	3 NS<br>2 SD<br>7 SI
P6 D7	**< 0.0001**	**< 0.0001**	**0.0035**	**0.0456**	0.2232	**0.0002**	**0.0074**	0.1272	**< 0.0001**	**< 0.0001**	0.0765	0.1000	4NS

ES -MMC													2 SD 6 SI
P6 D7 ES +MMC	**0.0034**	**< 0.0001**	**0.0029**	**0.0277**	**0.0382**	**< 0.0001**	**0.0280**	**0.0001**	**0.0001**	**< 0.0001**	0.0765	0.1000	2 NS 1 SD 9 SI
P9 D3 FBS -MMC	**< 0.0001**	**0.0084**	**0.0050**	0.1173	0.1300	0.5747	**< 0.0001**	0.4979	0.0691	**< 0.0001**	**< 0.0001**	0.1000	6 NS 4 SD 2 SI
P9 D3 FBS +MMC	**< 0.0001**	**< 0.0001**	**0.0035**	**0.0007**	**0.0119**	**0.0029**	**0.0002**	**0.0004**	**< 0.0001**	**< 0.0001**	**< 0.0001**	0.1000	1 NS 4 SD 7 SI
P9 D3 ES -MMC	0.562	**0.0027**	**0.0100**	0.1198	0.9314	**0.0042**	**< 0.0001**	**0.0218**	0.1571	**0.0003**	**< 0.0001**	0.1000	5 NS 4 SD 3 SI
P9 D3 ES +MMC	0.5572	**< 0.0001**	**0.0028**	0.2295	**0.0001**	**0.0003**	0.6879	**0.0002**	0.1000	0.1000	0.0722	0.1000	7 NS 1 SD 4 SI
P9 D5 FBS -MMC	**< 0.0001**	**0.0002**	0.1630	0.0747	0.1237	0.8828	**< 0.0001**	**0.0129**	0.4733	**< 0.0001**	0.0765	**0.0091**	6 NS 5 SD 1 SI
P9 D5 FBS +MMC	**< 0.0001**	**0.0001**	0.5467	**0.0009**	**0.0023**	**0.0040**	**< 0.0001**	**< 0.0001**	**0.0010**	**< 0.0001**	**< 0.0001**	0.1000	2 NS 5 SD 5 SI
P9 D5 ES	**0.0046**	**< 0.0001**	**0.0110**	0.7329	0.1901	**0.0007**	0.1142	0.4811	0.0715	**< 0.0001**	**0.0003**	0.1000	6 NS 3 SD

-MMC													3 SI
P9 D5<br>ES<br>+MMC	0.1069	**< 0.0001**	**0.0040**	0.1076	**0.0011**	**0.0002**	**0.0178**	**0.0008**	0.1000	0.1000	0.0765	**0.0098**	5 NS<br>2 SD<br>5 SI
P9 D7<br>FBS<br>-MMC	**< 0.0001**	**0.0014**	**0.0301**	0.0558	0.1387	0.1193	**< 0.0001**	0.2038	0.7000	**< 0.0001**	0.0765	0.1000	7 NS<br>3 SD<br>2 SI
P9 D7<br>FBS<br>+MMC	**< 0.0001**	**0.0006**	0.6127	**0.0152**	**0.0005**	**0.0003**	**0.0014**	**0.0012**	0.1000	**<0.0001**	**< 0.0001**	0.0765	3 NS<br>3 SD<br>6 SI
P9 D7<br>ES<br>-MMC	**0.0059**	**0.0451**	**0.0032**	0.0979	0.8981	**0.0027**	0.6304	0.8255	**0.0341**	**< 0.0001**	0.0765	0.1000	6 NS<br>1 SD<br>5 SI
P9 D7<br>ES<br>+MMC	0.1628	0.1290	**0.0080**	**0.0324**	**0.0004**	**< 0.0001**	0.6957	**< 0.0001**	0.1000	**0.0004**	**< 0.0001**	0.1000	5 NS<br>2 SD<br>5 SI

Supplementary Figure S1: Brightfield microscopy of eTCs as a function of serum, passage, MMC and time in culture.

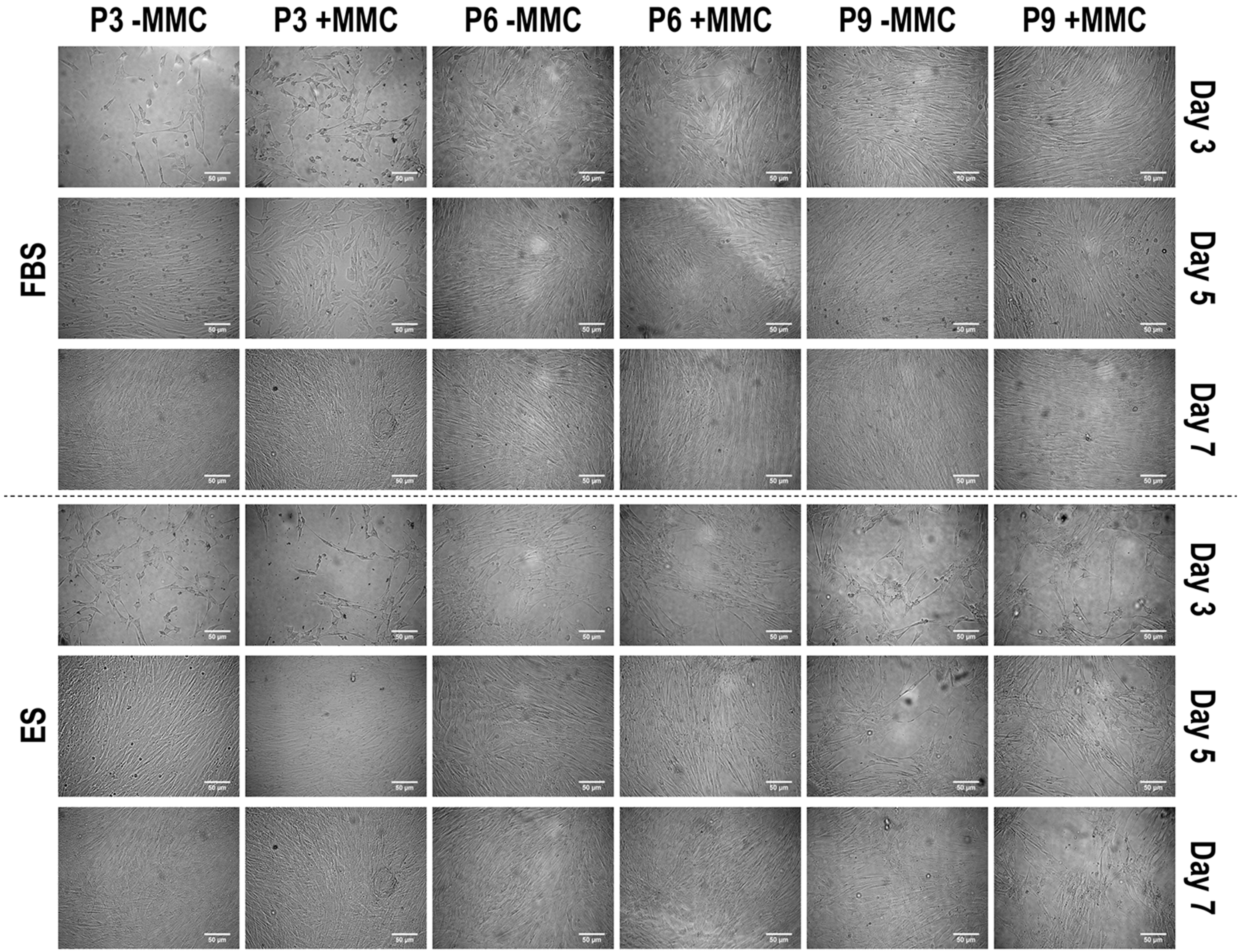

Supplementary Figure S2: eTC cell number (proliferation) as a function of serum, passage, MMC and time in culture. * indicates significant ($p < 0.05$) increase in comparison to cells at passage 3, day 3, in FBS and without MMC.

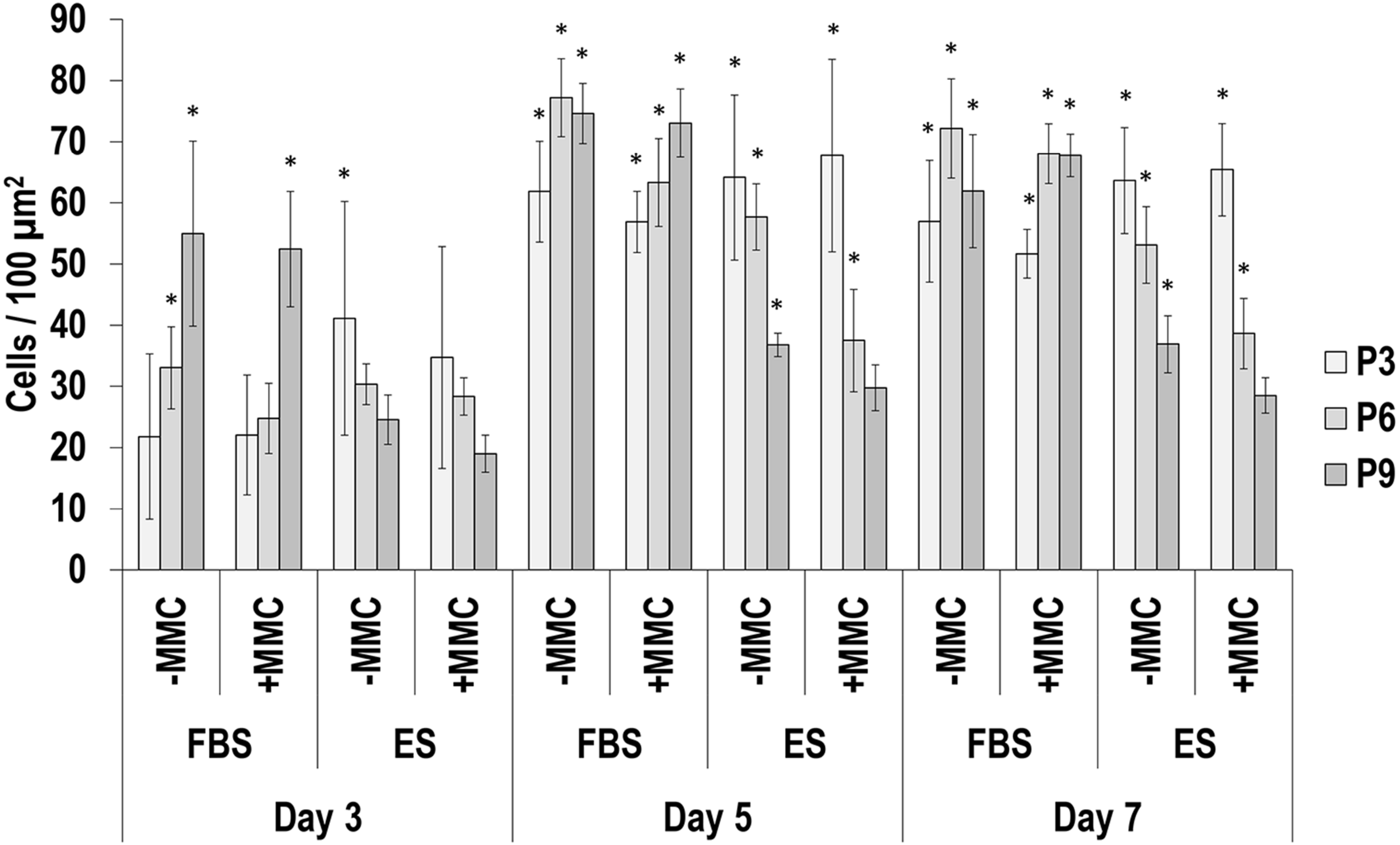

Supplementary Figure S3: eTC metabolic activity as a function of serum, passage, MMC and time in culture. * indicates significant ($p < 0.05$) increase in comparison to cells at passage 3, day 3, in FBS and without MMC. # indicates significant ($p < 0.05$) decrease in comparison to cells at passage 3, day 3, in FBS and without MMC.

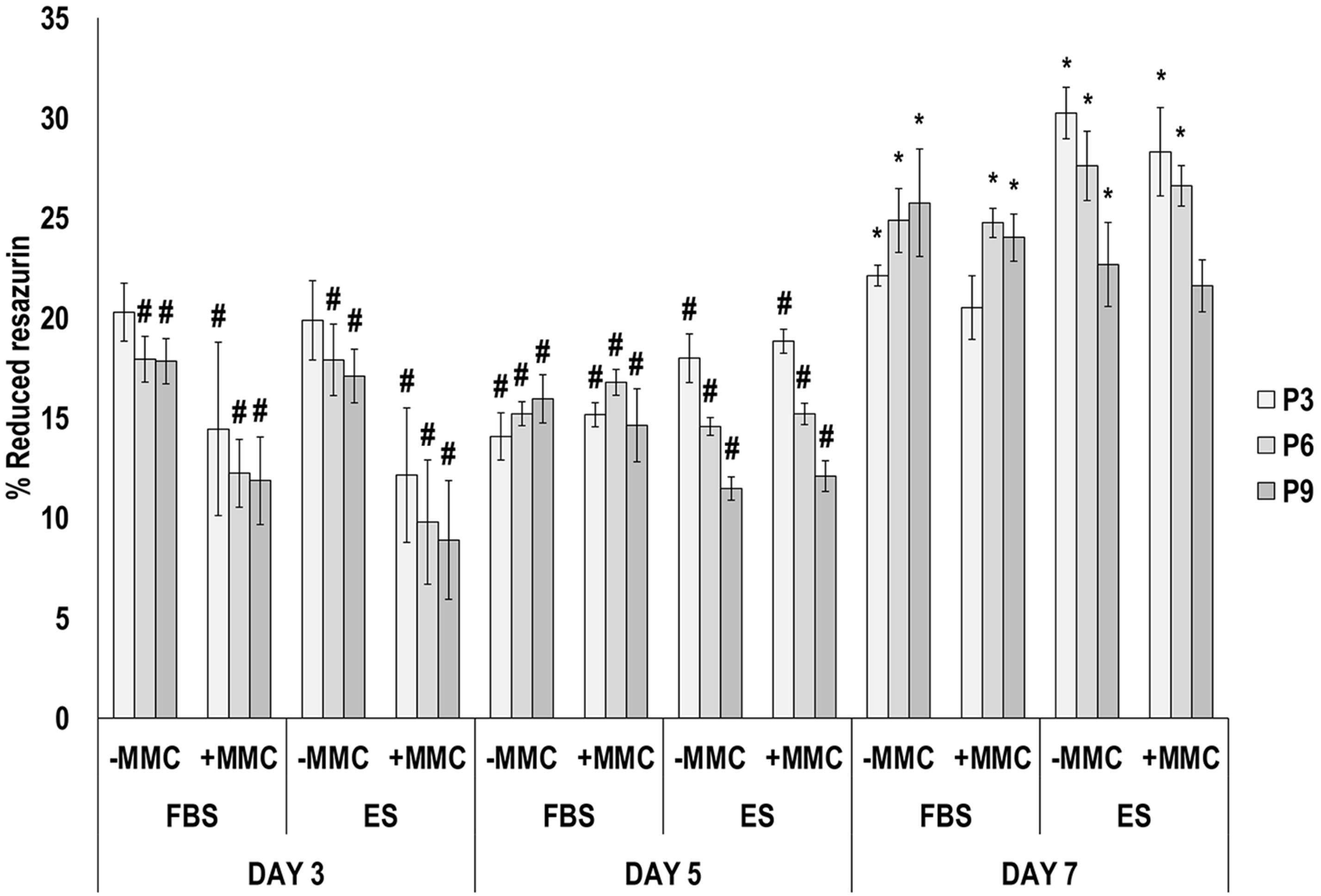

Supplementary Figure S4: eTC viability as a function of serum, passage, MMC and time in culture. * indicates significant ($p < 0.05$) increase in comparison to cells at passage 3, day 3, in FBS and without MMC. # indicates significant ($p < 0.05$) decrease in comparison to cells at passage 3, day 3, in FBS and without MMC.

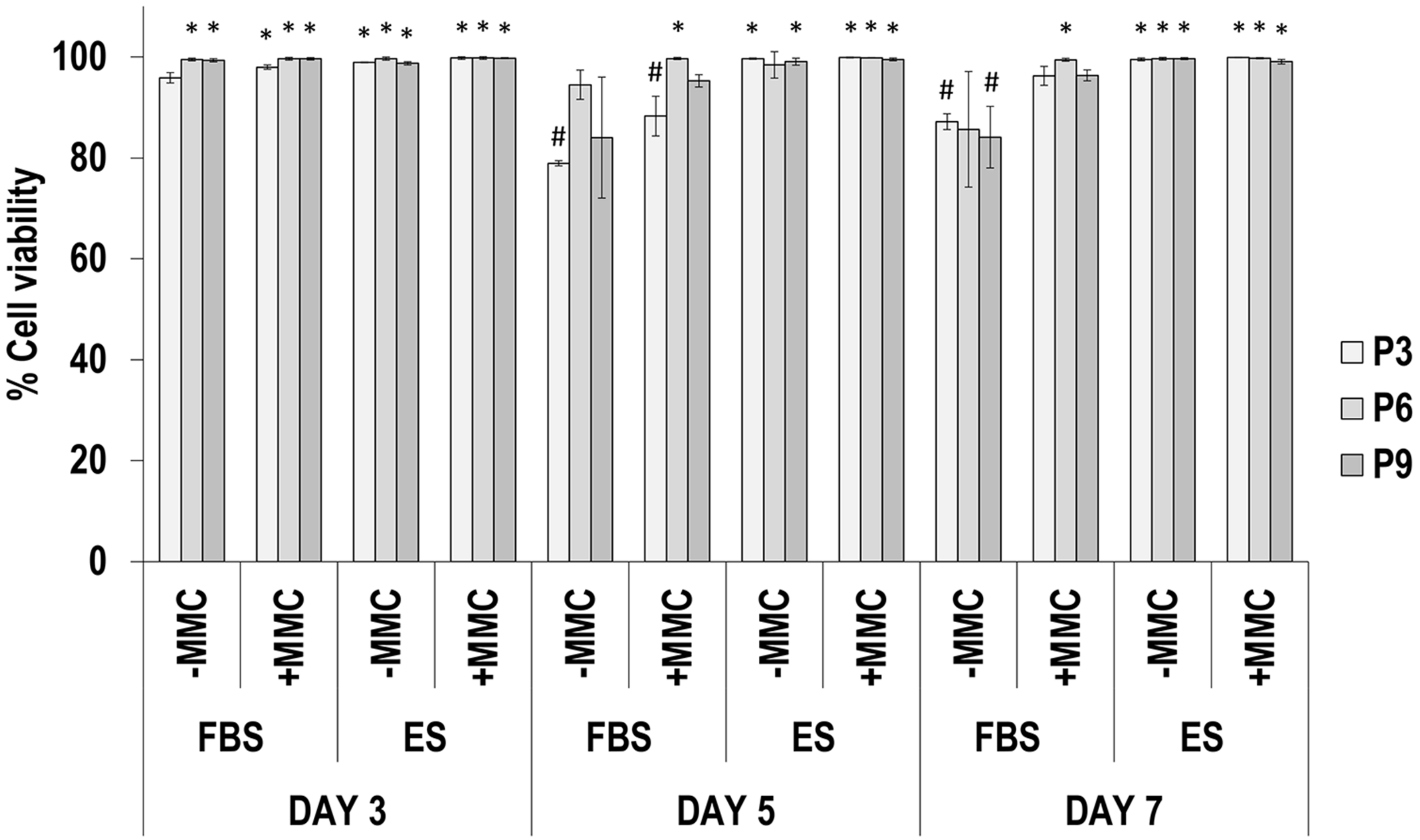

Supplement: Supplementary file 1 [file cells-11-01562-s001.zip › cells-1669769-supplementary.pdf]
